# Reference genes for *Eucalyptus* spp. under *Beauveria bassiana* inoculation and subsequently infestation by the galling wasp *Leptocybe invasa*

**DOI:** 10.1038/s41598-024-52948-x

**Published:** 2024-01-31

**Authors:** Matheus Martins Daude, Solange Aparecida Ságio, Jovielly Neves Rodrigues, Nívea Maria Pereira Lima, André Almeida Lima, Maíra Ignacio Sarmento, Renato Almeida Sarmento, Horllys Gomes Barreto

**Affiliations:** 1 Laboratory of Molecular Analysis (LAM), Life Sciences Department, Faculty of Medicine, Federal University of Tocantins, Palmas, TO Brazil; 2 Postgraduate Program in Biodiversity and Biotechnology, Rede Bionorte, Federal University of Tocantins, Palmas, TO Brazil; 3 Postgraduate Program in Digital Agroenergy, Federal University of Tocantins, Palmas, TO Brazil; 4 Postgraduate Program in Forest and Environmental Sciences, Federal University of Tocantins, Palmas, TO Brazil; 5 Agronomy Undergraduate Course, Federal University of Tocantins, Palmas, TO Brazil

**Keywords:** Biological techniques, Biotechnology, Genetics, Plant sciences

## Abstract

Relative gene expression analysis through RT-qPCR is an important molecular technique that helps understanding different molecular mechanisms, such as the plant defense response to insect pests. However, the use of RT-qPCR for gene expression analysis can be affected by factors that directly affect the reliability of the results. Among these factors, the appropriate choice of reference genes is crucial and can strongly impact RT-qPCR relative gene expression analyses, highlighting the importance in correctly choosing the most suitable genes for the success of the analysis. Thus, this study aimed to select and validate reference genes for relative gene expression studies through RT-qPCR in hybrids of *Eucalyptus tereticornis* × *Eucalyptus camaldulensis* (drought tolerant and susceptible to *Leptocybe invasa*) under conditions of inoculation by the *Beauveria bassiana* fungus and subsequent infestation by *L. invasa*. The expression level and stability of eleven candidate genes were evaluated. Stability was analyzed using the RefFinder tool, which integrates the geNorm, NormFinder, BestKeeper, and Delta-Ct algorithms. The selected reference genes were validated through the expression analysis of the transcriptional factor *EcDREB2* (dehydration-responsive element-binding protein 2). For all treatments evaluated, *EcPTB*, *EcPP2A*-1, and *EcEUC12* were the best reference genes. The triplets *EcPTB*/*EcEUC12*/*EcUBP6*, *EcPP2A-1*/*EcEUC12*/*EcPTB*, *EcIDH*/*EcSAND*/*Ecα-TUB*, *EcPP2A-1*/*Ecα-TUB*/*EcPTB*, and *EcPP2A-1*/*EcUPL7*/*EcSAND* were the best reference genes for the control plants, mother plants, plants inoculated with *B. bassiana,* plants infested with *L. invasa*, and plants inoculated with *B. bassiana* and subsequently infested with *L. invasa*, respectively. The best determined reference genes were used to normalize the RT-qPCR expression data for each experimental condition evaluated. The results emphasize the importance of this type of study to ensure the reliability of relative gene expression analyses. Furthermore, the findings of this study can be used as a basis for future research, comprising gene expression analysis of different eucalyptus metabolic pathways.

## Introduction

The *Eucalyptus* genus belongs to the *Myrtaceae* family and is composed by approximately 600 species and subspecies, being one of the main global sources of wood and widely cultivated for industrial use^1,2^. Worldwide, raw materials derived from the forestry sector are used in the production of various products, such as civil construction structures, furniture, paper, pharmaceutical, and cosmetic products, being also used for energy generation^3^. Globally, *Eucalyptus* spp. is the most extensively cultivated forest genus, with a planted area of around 25 million hectares^4^. Brazil stands out as the world's largest eucalyptus producer, with a cultivation area of more than 7 million hectares, which corresponds to 75.8% of the total area planted with trees, playing an important role in its economy, and generating a total revenue of more than 47 billion of dollars and 2 million jobs^5^. Thus, the economic and socioeconomic importance of forest resources has driven the growth of the sector, although the incidence of biotic factors, such as pests, still represents a challenge to be overcome^6,7^.

The eucalyptus gall wasp (*Leptocybe invasa*) is a biotic factor that affects the sustainability of eucalyptus plantations, as it causes serious damage through the induction of galls along the entire length of veins and on petioles of young leaves, and also in internodes of branch apices. Infestation by this pest can lead to devastating outcomes, such as rough appearance of plants, stunted growth, and in extreme cases, plant death^8–10^. To meet this challenge, it is essential to implement the best combination of integrated tools and techniques. Thus, studies related to plant-microorganism association, especially with endophytic organisms, are essential considering the great damage caused by the attack of insect pests, such as the eucalyptus gall wasp^11,12^. Endophytic fungi act symbiotically, triggering, for instance, local and/or systemic defenses^13^. The species *Beauveria bassiana* (Bals.) Vuill is an entomopathogenic fungus often used as a biopesticide in agricultural crops due to its environmental safety, not causing any risk to human health and displaying only minimal adverse effects on non-target organisms^12,14^. In *Eucalyptus*, the use of *B. bassiana* as an alternative biological agent to control the galling insect *L. invasa* has shown great potential for plant resistance to attack by this pest^12^.

The use of molecular techniques to study eucalyptus species, as well as studies aimed at biological control, has gained progressive attention due to the diverse possibilities they offer for a better comprehension of plant metabolism. The sequencing of the *Eucalyptus grandis* genome has enabled significant advances in molecular studies by providing information on genes related to metabolic pathways of great economic interest^15^. In this context, gene expression analysis is an important tool to better understand the molecular mechanisms behind different biological processes^16^ such as the defense response to *L. invasa* infestation under the presence of *B. bassiana.* Currently, several methods of gene expression analysis in plants are used^17^, with the quantitative Real-Time Polymerase Chain Reaction with reverse transcription (RT-qPCR) being one of the most common and widely used method due to its speed, high sensitivity, reproducibility, and precision in determining gene expression levels^18–20^. However, it is important to consider that the use of RT-qPCR for RNA quantification is prone to several factors that can directly affect the reliability of the results obtained^21^.

RNA (ribonucleic acid) integrity and quality, cDNA (complementary DNA) synthesis and amplification efficiencies, and the choice of reference genes are crucial factors that can strongly impact the results of RT-qPCR studies^20,22^. Therefore, to ensure reproducibility and minimize the variability of RT-qPCR assays, it is crucial to evaluate these parameters^21^. In particular, among the factors previously mentioned, the determination of reference genes is one of the most important aspects when using gene expression analysis by RT-qPCR^1^, since the efficiency of the technique depends on the validation of appropriate reference genes for accurate assay normalization and correction of nonspecific variations^23–25^. For this reason, the use of validated reference genes, based on their expression stability in different tissues and experimental conditions, is essential to guarantee the quality and reliability of the results^26^.

The use of reference genes for the normalization of RT-qPCR gene expression data is based on the fact that, in theory, their expression levels are constant, regardless of the tissues, developmental stages or physiological conditions of the evaluated species^17,27^. Thus, different genes involved in the maintenance of basic cellular functions such as cell division, growth and development, apoptosis, and other physiological processes are often used as reference genes due to their ability to be expressed in basically every cell of the organism^26^. Plants genes such as *ACT*, *TUB*, ribosomal RNA genes (*18S*, *rRNA*, *26S rRNA*), *GAPDH*, *UBQ*, *EF-1α*, *UBC*, and *PP2A* are commonly employed as reference genes^17,25^ However, different studies have shown that the expression of these genes may vary, leading to incorrect normalization of target genes^28,29^ and confirming that there is no universal reference gene^30–33^.

Different studies aiming to determine the best eucalyptus reference genes have been performed^1,2,34–39^. However, such studies are still necessary when specific genotypes, tissues or stress conditions are under study^40,41^. In this context, studies aimed to determine the best reference genes in eucalyptus under *L. invasa* infestation and inoculation of the *B. bassiana* fungus have not been carried out yet. Therefore, considering the importance of the RT-qPCR technique for gene expression studies and the need to validate suitable reference genes for specific conditions, the objective of this study was to select and validate reference genes for RT-qPCR studies in the *Eucalyptus. tereticornis* × *Eucalyptus. camaldulensis* hybrid (drought tolerant and susceptible to *L. invasa*) under the inoculation of *B. bassiana* and subsequently infestation by *L. invasa.*

## Results

### Identification and selection of target and reference genes

The literature review returned seven articles related to the selection of reference genes in *Eucalyptus* spp., from which four were included in this analysis. The inclusion criteria were based on the possibility of identifying the gene sequences described in each study. After selecting the articles, 12 reference genes were chosen, following the recommendation criteria of the study in which they were analyzed. In other words, the genes that showed the greatest expression stability in the samples evaluated in each study were selected. Thus, the following genes were chosen: *PP2A-1* (*Protein phosphatase 2A-2*), *SAND* (*SAND family, trafficking protein Mon1*), *UPL7* (*Ubiquitin-protein ligase 7*), *PTB* (*Polypyrimidine tract-binding protein 1*)^38^; *Actin-2* (*Actin 2*), *18srRNA* (*RNA ribosomal 18S*), *IDH* (*NADP-isocitrate dehydrogenase*)^34^, *α-TUB* (*α-Tubulin*), *UBC* (*Ubiquitin C*), *EF-1α* (*Elongation factor 1-α*)^37^, *EUC12* (*Putative RNA binding protein*), and *H2B* (*Histone H2B*)^36^.

In order to identify and select a target gene, gene expression levels of 155 genes from the AP2/EREBP superfamily (APETALA2/Ethylene Responsive Element Binding Protein) were analyzed during *L. invasa* infestation, using the gene expression graph generated by the ExHeatmap option, as described by Sundell et al.^42^. The transcriptional factor *DREB2* (dehydration-responsive element-binding protein) stood out from the analyzed genes, displaying high expression levels in *E. grandis* tissues subjected to *L. invasa* attack, being selected for the further analyses.

### Expression levels of the candidate reference genes

**Figure 1:**
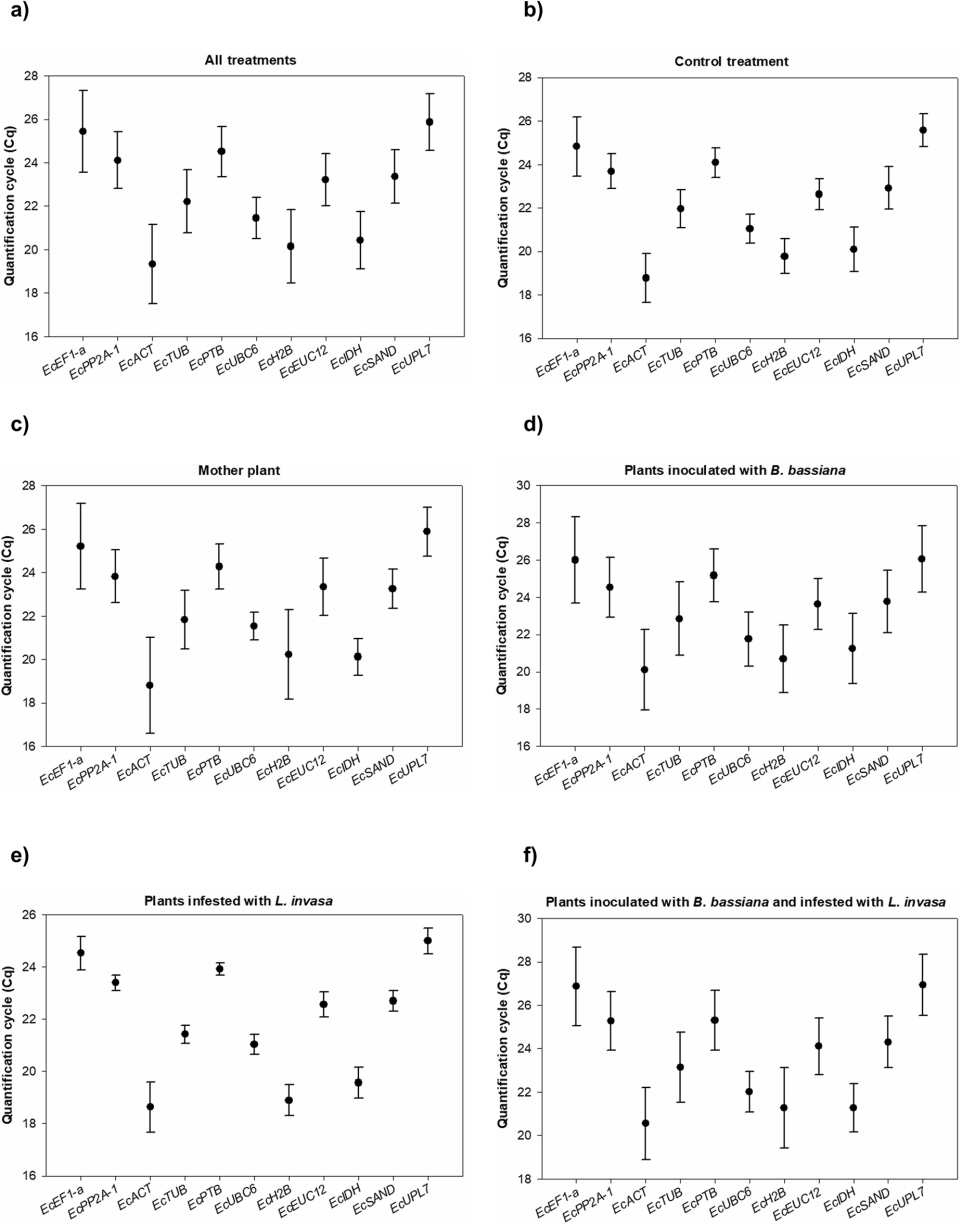
Candidate reference gene expression levels based on the Cq (Cycle of Quantification) data obtained from stem, leaves and leaf apex of eucalyptus plants under different treatments conditions (**a**), eucalyptus control plants (**b**), eucalyptus mother plants (**c**), eucalyptus plants inoculated with *B. bassiana* (**d**), eucalyptus plants infested with *L. invasa* (**e**), and eucalyptus plants inoculated with *B. bassiana* and infested with *L. invasa* (**f**). Vertical bars represent the standard deviation, and the black dots represent the mean Cq values.

The expression analyses of the candidate reference genes in all treatments (Fig. 1A) indicated a wide variation in the Cqs average values, with the minimum and maximum values of 19.33 and 25.87, respectively. When each treatment was separately analyzed, it was possible to observe a similar expression pattern to the one observed when all treatments are analyzed together (Fig. 1A). The Cq average values in the control treatment (Fig. 1B), in mother plants (Fig. 1C), in plants inoculated with *B. bassiana* (Fig. 1D), in plants infested with *L. invasa* (Fig. 1E), and in plants inoculated with *B. bassiana* and infested with *L. invasa* (Fig. 1F) ranged from 18.77, 18.79, 18.64, 20.10, and 20.55 (lowest value) to 25.58, 25.88, 24.99, 26.06, and 26.94 (highest value), respectively.

The Cq average values for all genes revealed that, regardless of the evaluated condition, the three genes with the highest and lowest expression levels were the same. When comparing the average Cq values of each treatment (Fig. 1), *EcACT*, *EcH2B*, and *EcIDH* showed the highest expression levels, with values varying from 18.64 to 21.27. In contrast, *EcUPL7*, *EcEF1-α*, and *EcPTB* displayed the lowest expression levels, with Cq average values ranking from 23.91 to 26.94.

### Expression stability of the candidate reference genes

**Figure 2:**
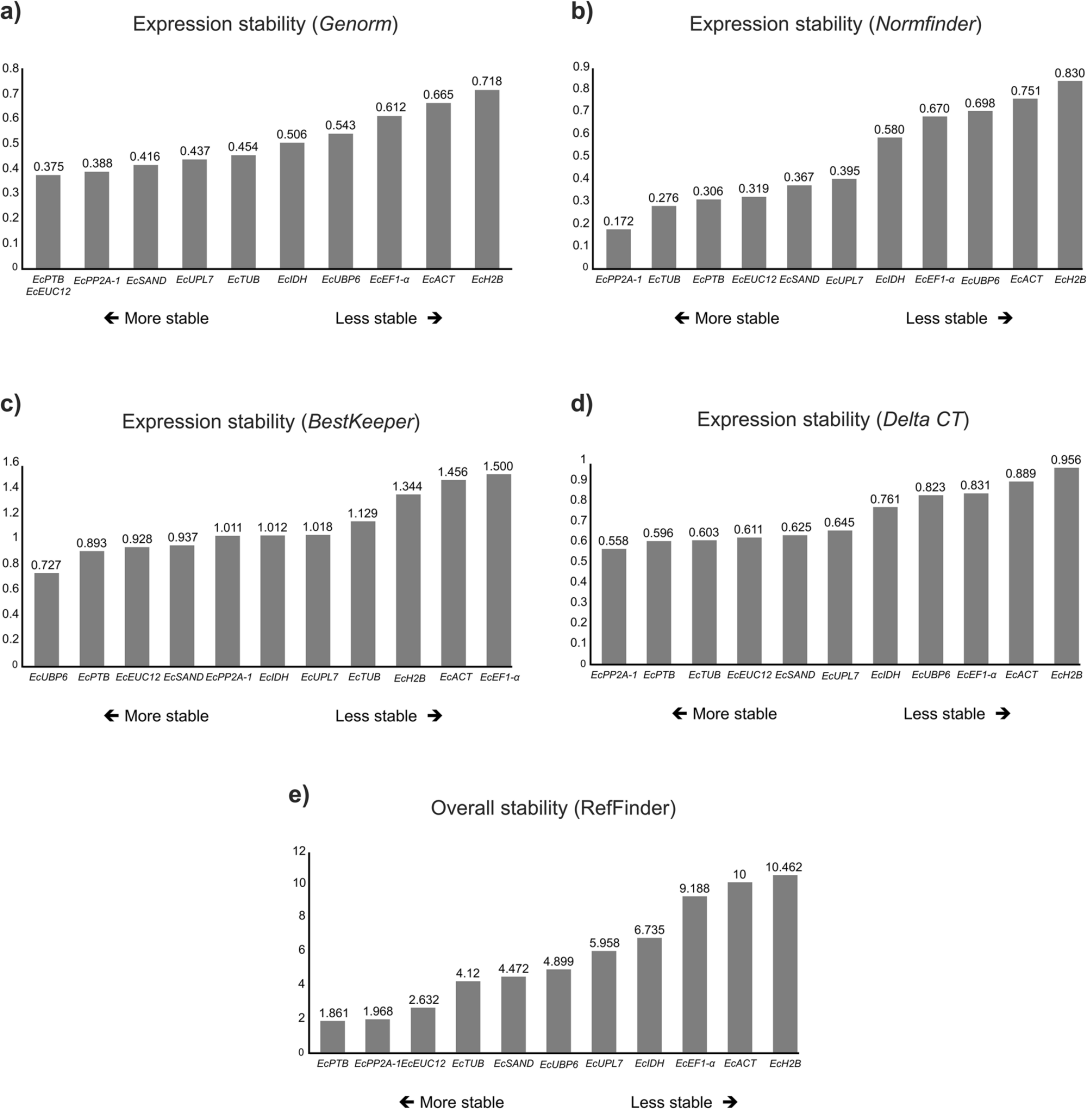
Ranking of the candidate reference genes according to their stability value calculated by the geNorm (**a**), NormFinder (**b**), BestKeeper (**c**), Delta-Ct (**d**), and RefFinder (**e**) algorithms using the Cq data (Cycle of Quantification) obtained from all five treatments (Control plants; Mother plants; Plants infested with *L. invasa*; Plants inoculated with *B. bassiana*; Plants infested with *L. invasa* and inoculated with *B. bassiana*).

Tables 1, 2, 3, 4 and 5, along with Fig. 2 describe the expression stability analysis of the candidate reference genes using the geNorm, NormFinder, BestKeeper, Delta-Ct, and RefFinder algorithms. The data presented in the previously mentioned tables comprise the analysis of gene stability in 5 sample sets, and the Cqs of all genes and conditions evaluated can be found in Tables (Supplementary S1), making it possible to combine several other sample sets.

**Table 1:** Ranking of the reference gene candidates generated according to their stability values calculated by the geNorm, NormFinder, BestKeeper, Delta-Ct, and Refiner algorithms using the Cq (Cycle of Quantification) data obtained from stem, leaves, and leaf apex of eucalyptus control plants.

Gene	geNorm		NormFinder		BestKeeper		Delta-Ct		RefFinder	
	M stability value	Ranking	SV stability value	Ranking	SD stability value	Ranking	ΔCt stability value	Ranking	Overall stability value	Overall ranking
*EcPTB*	0.172	1	0.057	1	0.549	2	0.490	1	1.189	1
*EcEUC12*	0.172	1	0.113	2	0.566	3	0.496	2	1.861	2
*EcUBP6*	0.331	4	0.466	5	0.535	1	0.627	5	3.344	3
*EcPP2A-1*	0.233	2	0.149	3	0.617	5	0.504	3	3.409	4
*Ecα-TUB*	0.265	3	0.237	4	0.656	6	0.537	4	4.427	5
*EcUPL7*	0.349	5	0.467	6	0.580	4	0.628	6	5.422	6
*EcSAND*	0.410	6	0.475	7	0.850	8	0.669	7	7.238	7
*EcACT*	0.493	7	0.593	8	0.937	10	0.750	8	8.459	8
*EcIDH*	0.554	8	0.658	9	0.913	9	0.803	9	9.000	9
*EcH2B*	0.661	10	0.827	11	0.672	7	0.926	11	9.825	10
*EcEF1-α*	0.602	9	0.722	10	1.239	11	0.844	10	10.241	11

**Table 2:** Ranking of the reference gene candidates generated according to their stability values calculated by the geNorm, NormFinder, BestKeeper, Delta-Ct, and RefFinder algorithms using the Cq (Cycle of Quantification) data obtained from stem, leaves, and leaf apex of eucalyptus mother plants.

Gene	geNorm		NormFinder		BestKeeper		Delta-Ct		RefFinder	
	M stability value	Ranking	SV stability value	Ranking	SD stability value	Ranking	M stability value	Ranking	SV stability value	Ranking
*EcPP2A-1*	0.292	1	0.146	2	1.011	6	0.626	1	1.861	1
*EcEUC12*	0.292	1	0.120	1	1.089	7	0.639	3	2.141	2
*EcPTB*	0.310	2	0.225	4	0.888	4	0.628	2	3.130	3
*Ecα-TUB*	0.345	3	0.175	3	1.198	8	0.680	4	4.427	4
*EcUBP6*	0.500	7	0.826	9	0.458	1	0.932	8	4.899	5
*EcUPL7*	0.383	4	0.428	5	0.936	5	0.710	5	5.000	6
*EcSAND*	0.426	5	0.493	6	0.754	3	0.751	6	5.045	7
*EcIDH*	0.457	6	0.649	7	0.682	2	0.805	7	5.118	8
*EcEF1-α*	0.616	8	0.758	8	1.735	9	0.970	9	8.739	9
*EcACT*	0.730	9	1.071	10	1.839	11	1.202	10	10.241	10
*EcH2B*	0.847	10	1.269	11	1.764	10	1.372	11	10.741	11

**Table 3:** Ranking of the reference gene candidates generated according to their stability values calculated by the geNorm, NormFinder, BestKeeper, Delta-Ct, and RefFinder algorithms using the Cq (Cycle of Quantification) data obtained from stem, leaves, and leaf apex of eucalyptus plants inoculated with *B. bassiana*.

Gene	geNorm		NormFinder		BestKeeper		Delta-Ct		RefFinder	
	M stability value	Ranking	SV stability value	Ranking	SD stability value	M stability value	Ranking	SV stability value	Ranking	SD stability value
*EcIDH*	0.197	1	0.152	1	1.634	8	0.417	2	2.000	1
*EcSAND*	0.276	3	0.161	2	1.420	5	0.405	1	2.515	2
*Ecα-TUB*	0.197	1	0.229	4	1.659	9	0.441	4	3.464	3
*EcUPL7*	0.258	2	0.185	3	1.533	7	0.423	3	3.708	4
*EcPTB*	0.366	6	0.405	7	1.219	2	0.505	7	5.118	5
*EcPP2A-1*	0.330	5	0.261	6	1.393	4	0.457	5	5.180	6
*EcEUC12*	0.403	8	0.499	9	1.114	1	0.574	9	5.196	7
*EcH2B*	0.305	4	0.244	5	1.529	6	0.460	6	5.477	8
*EcUBP6*	0.385	7	0.454	8	1.264	3	0.537	8	6.260	9
*EcACT*	0.466	9	0.619	10	1.806	10	0.689	10	10.000	10
*EcEF1-α*	0.511	10	0.658	11	1.970	11	0.716	11	11.000	11

**Table 4:** Ranking of the reference gene candidates generated according to their stability values calculated by the geNorm, NormFinder, BestKeeper, Delta-Ct, and RefFinder algorithms using the Cq (Cycle of Quantification) data obtained from stem, leaves, and leaf apex of eucalyptus plants infested with *L. invasa*.

Gene	geNorm		NormFinder		BestKeeper		Delta-Ct		RefFinder	
	M stability value	Ranking	SV stability value	Ranking	SD stability value	Ranking	M stability value	Ranking	SV stability value	Ranking
*EcPP2A-1*	0.189	1	0.072	1	0.224	2	0.431	2	1.414	1
*Ecα-TUB*	0.189	1	0.094	2	0.276	3	0.415	1	1.565	2
*EcPTB*	0.221	2	0.179	3	0.182	1	0.455	3	2.280	3
*EcEUC12*	0.272	3	0.246	4	0.375	6	0.493	4	4.427	4
*EcSAND*	0.317	4	0.326	5	0.288	4	0.516	5	4.729	5
*EcUPL7*	0.345	5	0.359	6	0.414	8	0.545	6	6.447	6
*EcUBP6*	0.421	7	0.493	8	0.328	5	0.618	8	7.113	7
*EcEF1-α*	0.382	6	0.374	7	0.523	10	0.553	7	7.653	8
*EcIDH*	0.472	8	0.675	10	0.399	7	0.752	10	8.909	9
*EcH2B*	0.521	9	0.618	9	0.503	9	0.724	9	9.240	10
*EcACT*	0.575	10	0.749	11	0.767	11	0.817	11	11.000	11

**Table 5:** Ranking of the reference gene candidates generated according to their stability values calculated by the geNorm, NormFinder, BestKeeper, Delta-Ct, and RefFinder algorithms using the Cq (Cycle of Quantification) data obtained from stem, leaves, and leaf apex of eucalyptus inoculated with *B. bassiana* and plants infested with *L. invasa*.

Gene	geNorm		NormFinder		BestKeeper		Delta-Ct		RefFinder	
	M stability value	Ranking	SV stability value	Ranking	SD stability value	M stability value	Ranking	SV stability value	Ranking	SD stability value
*EcPP2A-1*	0.264	1	0.151	2	1.245	5	0.477	2	2.115	1
*EcUPL7*	0.291	3	0.124	1	1.262	6	0.476	1	2.213	2
*EcSAND*	0.264	1	0.212	4	1.091	3	0.490	3	2.449	3
*EcEUC12*	0.274	2	0.210	3	1.220	4	0.493	4	3.464	4
*EcIDH*	0.349	4	0.438	7	0.994	2	0.604	7	4.705	5
*EcUBP6*	0.609	10	0.870	11	0.782	1	0.931	11	6.040	6
*Ecα-TUB*	0.424	6	0.395	5	1.503	8	0.589	5	6.117	7
*EcPTB*	0.387	5	0.405	6	1.274	7	0.590	6	6.236	8
*EcACT*	0.451	7	0.447	8	1.561	9	0.612	8	8.239	9
*EcEF1-α*	0.499	8	0.561	9	1.571	10	0.683	9	9.240	10
*EcH2B*	0.537	9	0.666	10	1.605	11	0.753	10	10.241	11

### Control plants

In control plants (Table 1), the geNorm, NormFinder, and Delta-Ct algorithms indicated that *EcPTB*, *EcEUC12*, and *EcPP2A-1* were the most stable genes. The BestKeeper algorithm, on the other hand, found that *EcUBP6*, *EcPTB*, and *EcEUC12* were most stable in this treatment. In terms of the general classification provided by the RefFinder algorithm, *EcPTB*, *EcEUC12*, and *EcUBP6* were the best reference genes. In relation to the least stable genes, *EcH2B*, *EcEF1-α*, and *EcIDH* were identified by the geNorm, NormFinder, and Delta-Ct algorithms as the least stable genes. BestKeeper classified *EcEF1-α*, *EcACT*, and *EcIDH* as the most variable genes and RefFinder considered *EcEF1-α*, *EcH2B*, and *EcIDH* as the genes with the lower stability values.

### Mother plants

The stability values of the candidate reference genes for the mother plants (Table 2) presented different results among the algorithms analyzed. *EcPP2-A*, *EcEUC12*, and *EcPTB* were indicated as the most stable genes by geNorm and Delta-Ct algorithms, while *EcEUC12*, *EcPP2-A*, and *Ecα-TUB* were classified as the most stable by NormFinder. On the other hand, according to BestKeeper, *EcUBP6*, *EcIDH*, and *EcSAND* are most stable genes for mother plants and for RefFinder *EcPP2-A*, *EcEUC12*, and *EcPTB* displayed the higher expression stability. About the least stable genes, all algorithms identified *EcH2B*, *EcACT*, and *EcEF1-α* as the worst (least stable) genes, except for NormFinder that, instead of *EcEF1-α*, classified *EcUBP6* among the three least stable genes.

### Plants inoculated with *B. bassiana*

For the plants inoculated with the *B. bassiana* fungus, the expression stability analysis revealed significant differences among the five algorithms used (Table 3). The geNorm listed EcIDH, *Ecα-TUB*, and *EcUPL7* as the most stable genes, while NormFinder considered *EcIDH*, *EcSAND*, and *EcUPL7*. BestKeeper classified *EcEUC12*, *EcPTB*, and *EcUBP6* as the most stable genes and for Delta-Ct, on the other hand, *EcSAND*, *EcIDH*, and *EcUPL7* showed the best stability levels. Finally, RefFinder, which takes into account the results of all algorithms, classified *EcIDH*, *EcSAND*, and *Ecα-TUB* as the most stable genes. In relation to the genes with relatively lower stability, geNorm, NormFinder, and Delta-Ct classified *EcEF1-α*, *EcACT*, and *EcEUC12* as the least stable genes. Similarly, the BestKeeper algorithm also identified *EcEF1*-α and *EcACT* as the genes with lower stability levels and differed only in the inclusion of *Ecα-TUB*, instead of *EcEUC12*, when compared to the previously mentioned algorithms. For the overall ranking defined by RefFinder, *EcEF1-α*, *EcACT*, and *EcUBP6* were found to be the least stable genes.

### Plants infested with *L. invasa*

When eucalyptus plants were under *L. Invasa* infestation, the four algorithms used consistently indicated the same set of genes as the most stable ones: *EcPP2A-1*, *Ecα-TUB*, and *EcPTB* (Table 4). Similarly, these algorithms also identified the same genes as the least stable ones: *EcACT*, *EcH2B*, and *EcIDH*, except for BestKeeper, which indicated the *EcEF1-α* instead of *EcIDH*. The only difference, in both cases, was the position in which the genes were classified (Table 4).

### Plants inoculated with *B. bassiana* and infested with *L. invasa*

Similarities and divergences could be found for eucalyptus plants under *B. bassiana* inoculation and *L. invasa* infestation (Table 5). For the most stable genes, geNorm pointed out the genes *EcPP2A-1*, *EcSAND*, and *EcEUC12* as the best ones. NormFinder identified *EcUPL7*, *EcPP2A-1* and, *EcEUC12* as the most stable genes, while BestKeeper presented a slightly different classification, classifying *EcUBP6*, *EcIDH*, and *EcSAND* as the genes with higher expression stability. The Delta-Ct and the RefFinder considered the same set of genes, *EcPP2A-1*, *EcUPL7*, and *EcSAND*, as the most stable in terms of expression, although in different positions (Table 5). For the least stable genes, the geNorm, NormFinder, and DeltaCT algorithms indicated *EcUBP6*, *EcH2B*, and *ECEF1-α* as the genes displaying higher expression variability. On the other hand, BestKeeper ranked *EcH2B*, *EcEF1-α*, and *EcACT* as the least stable genes, just like RefFinder (Table 5).

### Overall treatment analysis

Cq values from the five evaluated treatments were combined to analyze the overall expression stability of the candidate reference genes (Fig. 2). *EcPP2A-1*, *EcPTB*, and *Ecα-TUB* were defined by NormFinder and DeltaCt algorithms as the most stable genes. geNorm indicated *EcPTB*, *EcEUC12*, and *EcPP2A-1* as the genes with higher expression stability, while BestKepper listed *EcUBP6*, *EcPTB*, and *EcEUC12*. RefFinder classified *EcPTB*, *EcPP2A-1*, and *EcEUC12* as the most stable reference genes when every treatment is analyzed at the same time. Regarding the least stable genes, the geNorm, BestKepper, DeltaCt, and RefFinder algorithms indicated the same set of genes, *EcH2B*, *EcACT*, and *EcEF1-α*, as those with higher expression variability, while for NormFinder, *EcH2B*, *EcACT*, and *EcUBP6* were classified as least stable genes.

### Reference gene validation

**Figure 3:**
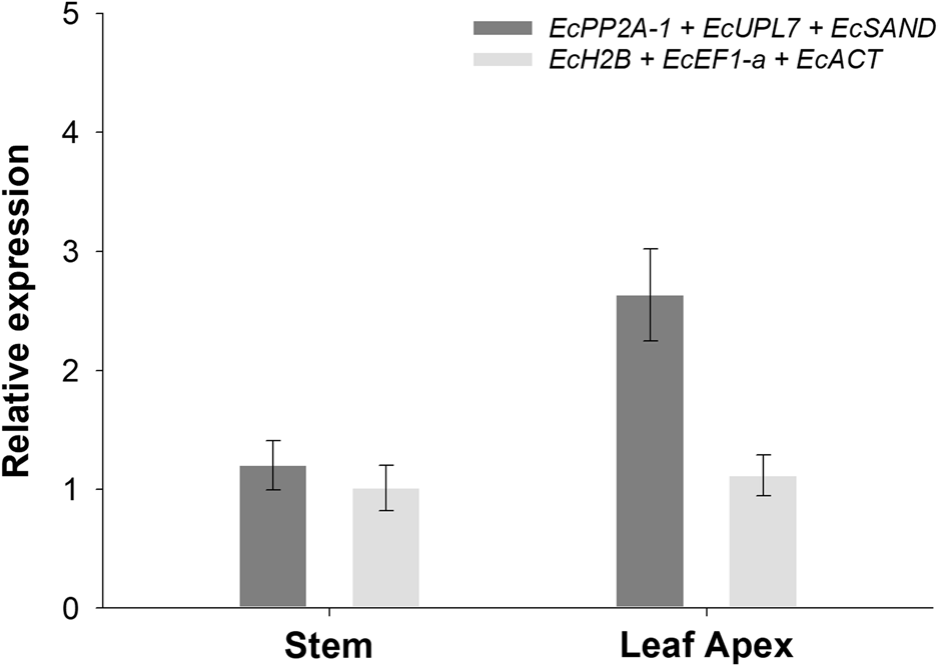
*EcDREB2* expression pattern, normalized with the most (*EcPP2A-1*, *EcULP7* and *EcSAND*) and least (*EcH2B*, *EcEF1-α* e *EcACT*) stable reference genes, according to the RefFinder algorithm, in stem and leaf Apex from eucalyptus plants submitted to *L. invasa* infestation and *B. Bassiana* inoculation. Columns represent the fold different in gene expression in relation to a calibrator sample (stem). Expression levels were obtained from three biological replicates and the error bars represent the standard error among these replicates.

The impact of choosing different reference genes was observed by analyzing the relative expression of *EcDREB2* (Fig. 3). The expression profile of the *EcDREB2* obtained from plants inoculated with the fungus *B. bassiana* and infested with the wasp *L. invasa* showed that significant differences can occur depending on the reference genes chosen for the normalization process.

When *EcDREB2* was normalized by using the most stable reference genes according to RefFinder (*EcPP2A-1*, *EcULP7*, and *EcSAND*) its expression was more than two times higher in leaf apex when compared to stem tissues (Fig. 3). However, when normalized by the least stable reference genes (*EcH2B*, *EcEF1-α*, and *EcACT*) there was no difference in *EcDREB2* expression between these tissues.

## Discussion

RT-qPCR analysis is universally accepted as a robust tool for quantifying gene expression levels^33,43^. However, it is important to highlight that the calculation used to measure the gene expression levels requires an essential step that is data normalization^44^. Normalization is necessary to correct experimental variations, such as differences in sample collection, total RNA extraction, cDNA synthesis, and procedures inherent to the RT-qPCR technique itself, guaranteeing the precision, reproducibility, and reliability of the results^45,46^. In this context, a common strategy employed is the use of reference genes as internal normalization standards. This approach is widely recognized as one of the most efficient for correcting nonspecific variations and achieving accurate normalization of gene expression data by RT-qPCR^25,47^.

The formulas used to calculate gene expression levels by RT-qPCR, such as those described by Livak and Schmittgen^48^ and Pfaffl^49^, necessarily require the use of reference genes. Therefore, their appropriate selection, as performed in this research, is a fundamental preliminary step in gene expression studies, since the use of inappropriate reference genes can significantly compromise the accuracy and interpretation of the results^33,50^. In this sense, several studies have been carried out, in different organisms and experimental conditions, with the aim of selecting and validating the best reference genes for RT-qPCR gene expression analyses^51–54^.

The results obtained in the present study provides the most suitable reference genes for the normalization of RT-qPCR data in eucalyptus plants under the inoculation by *B. bassiana* and *L. invasa* infestation. Although several studies related to the selection of reference genes in eucalyptus have been performed so far^1,2,34–37^, this is the first time that such analysis is conducted in *Eucalyptus* (*E. tereticornis* × *E. camaldulensis* hybrid) plants under *B. bassiana* inoculation and *L. invasa* infestation.

### Expression levels of all candidate reference genes

Selection of suitable reference gene requires meeting several criteria, including: (1) to be expressed at different developmental stages, physiological conditions, and tissues; (2) to display stable expression levels among different tissues and developmental, physiological and environmental conditions; (3) to display moderate to high expression levels (Cq value between 15 and 30); (4) to display the ability to reflect variations in the quantity and quality of RNA^47,55^. These criteria are essential to ensure accurate and reliable choice of a reference gene.

The results found here demonstrated that the maximum and minimum Cq values from all tissues and experimental conditions were within the range recommended in the literature (minimum of 19.33 and maximum of 25.87)^55^. Furthermore, when comparing the data obtained in this study with other studies carried out in eucalyptus that used the same analysis approach, a similar Cq variation was observed among the candidate reference genes. In the study conducted by Boava et al.^35^, the maximum and minimum Cq values of the 13 reference genes varied from 16 to 27. De Almeida et al.^36^ analyzed the expression of 11 candidate reference genes and obtained minimum and maximum Cq values of 12 and 25, a difference of 13 cycles. In the study conducted by Fernández et al.^37^, 10 genes were evaluated and the Cq variation among them was of approximately 7 cycles (Cqs from 21 to 28). In our data, the minimum and maximum Cq values among all genes evaluated varied from 18 to 27.

These results are in accordance with the variations observed in the literature and also with two of the essential criteria highlighted by Ling et al.^55^ and Mughal et al.^47^ for the selection of adequate reference genes: (1) to be expressed at different developmental stages, physiological conditions, and tissues; (2) to display moderate to high expression levels (Cq value between 15 and 30). This contributes to the reliability of the analyses performed in this study and reinforces the importance of carefully evaluating gene expression levels when aiming to select the most appropriate reference genes for normalizing RT-qPCR gene expression data. Furthermore, this analysis has been widely explored in several studies with similar objectives^51,54,56–58^.

### Reference gene expression stability

Reference genes have been extensively used for the normalization of RT-qPCR gene expression data, as they are assumed to be expressed at constant levels, regardless of the tissues under analysis, experimental treatment, developmental stages or physiological conditions of the species evaluated^28,56^. Thus, the use of genes involved in basal metabolism and structural integrity of the cell as reference genes is based on the general assumption that their expression levels are stable, independent of environmental/experimental conditions and cell type^59^. Therefore, genes that play essential roles in maintaining basic cellular functions, such as growth and development processes, apoptosis, cell division, and other physiological processes, are commonly used as reference genes, due to their ability to be expressed in all cells of an organism^26^.

*ACT*, *GAPDH*, *EF1-α*, *TUB*, *UBC*, and *PP2A* are often used as reference genes for the normalization of RT-qPCR studies^25^. However, several studies have demonstrated that the expression of many commonly used reference genes can significantly vary their expression pattern, and this may lead to inadequate normalization of target genes^19,28,29,44,60^. This reinforces that no universal reference gene has been identified, since several studies have shown that none of the reference genes tested so far can be used across different species, environmental conditions, developmental stages, or different tissues^30–32,61^. Therefore, evaluating reference gene stability in different tissues, developmental, physiological, and environmental conditions is one of the fundamental criteria during the process of selecting the most suitable reference genes^47,55^.

Genes commonly used and described in literature as adequate reference genes, such as *ACT*, *TUB*, *PP2A*, *UBC*, and *EF1-α*, were evaluated in this study and the results confirmed that the selection of more stable reference genes may vary depending on the experimental conditions. This pattern has also been observed in other eucalyptus reference genes studies^1,2,35^. Thus, since the ideal reference gene must present relatively stable expression levels in different cultivars, tissues and conditions^47,55^, the results described in the literature highlight the challenge of identifying suitable genes for different conditions and the importance of validating reference genes.

The algorithms used in this study, geNorm^59^, NormFinder^62^, BestKeeper^63^, Delta-Ct^64^, and RefFinder^65,66^ have been commonly used to evaluate the stability of reference genes in plants^19,25,54^, animals^67–69^ and microorganisms^70–72^. They are used to calculate the stability of gene expression using different mathematical models. Consequently, when analyzing the results among these algorithms, divergences can be observed in the establishment of the most stable genes^60^. In order to increase the reliability of the reference gene selection process, at least two different algorithms should be used to select the most stable reference genes^73^, since there may be some variation in the selected genes among them^1,28,44,60,74^. For this reason, RefFinder stands as an interesting alternative, instead of using these algorithms individually, since it uses the individual results obtained by each algorithm to perform a reanalysis and generate an overall classification of the most stable genes^66^. The RefFinder tool has been widely used in studies aimed at determining the best reference genes^51,54,56,74^.

In the present study, the Cq data of all candidate reference genes were submitted to a stability analysis using RefFinder, which allowed the identification of the genes that showed the greater stability in each treatment and enabled the observation of some similarities with other studies conducted eucalyptus plants. In our results, *SAND* gene was one of the most stable genes in plants inoculated with *B. bassiana* (Table 3) and plants inoculated with *B. bassiana* and infested with *L. invasa* (Table 5). Moura et al.^1^, for example, showed that *SAND* was one of the most stable genes under different conditions and different eucalyptus species. Similarly, De Almeida et al.^36^ defined the *SAND* gene as the most stable genes according to the geNorm algorithm during in vitro adventitious rooting of *Eucalyptus globulus* Labill. The *TUB* gene expression pattern in plants inoculated with *B. bassiana* (Table 3) and plants infested with *L. invasa* (Table 4) was one of the most stable when compared to the other analyzed genes, similar to the study conducted by Fernández et al.^37^, where *TUB* was also one of the best reference genes across different acclimation and de-acclimation treatments of *E. globulus*. Considering all treatments evaluated (Fig. 2), control plants (Table 1), and mother plants (Table 2), one of the best reference genes analyzed was *EUC12*. De Almeida et al.^36^ analyzed in vitro adventitious rooting in *E. globulus* and also found that *EUC12* displayed one of the most stable expression patterns among the genes analyzed according to the geNorm algorithm*.* It is important to highlight that the comparison between the best genes defined in other studies must consider the species, tissues and treatments analyzed. However, the fact that, in some cases, the same genes from different studies are defined as most stable ones suggests a high expression stability of them in different eucalyptus species, conditions and tissues.

### Reference gene validation

Plants are organisms capable of sensing environmental changes and adjusting their physiological state to adapt to new conditions. Successful plant defense responses depend on the timely accumulation of defense compounds, which are activated according to the nature of the pathogen. In this context, pathogen attack can lead to changes in gene expression and metabolic modifications that allow the establishment of an efficient defense response. Transcription factors play a crucial role in the plant innate immunity. Specifically, ethylene response factors (*ERF*) are important integrators of hormonal pathways and play a direct role in the transcriptional regulation of several defense genes activated by jasmonate/ethylene^75^.

The AP2/EREBP superfamily is one of the largest groups of plant-specific transcription factors^76^. Genes belonging to this superfamily play a crucial role in plant development, as well as in their ability to tolerate biotic, and abiotic stresses^77^. These genes are essential for plant growth and the development and response to various stresses, such as extreme temperatures, drought, high salinity, and pathogen infection. Furthermore, they are involved in several hormonal signaling pathways, including abscisic acid, ethylene, cytokinins, and jasmonates^76,78^.

The DREB (drought-responsive binding elements) genes belong to the AP2/EREBP superfamily and are important regulators of the response to abiotic stress^79^. Particularly, *DREB2* is mainly involved in dehydration/heat tolerance^80^. Although its main function is associated with the response to these stresses, *DREB2* also displays high expression levels in tissues under insect attack^42^. However, studies investigating the transcriptional expression of these genes in response to galling insect attacks are still scarce. Therefore, in the present work, the relative expression of *EcDREB2* was analyzed, allowing us to observe its expression in tissues subjected to *B. bassiana* inoculation and gall wasp attack. In comparison with the data available in the tool developed by Sundell et al.^42^, it could be observed that the *EcDREB2* expression pattern, when the RT-qPCR data were normalized with the best reference genes (Fig. 3), is similar, showing high expression levels in plants infested with *L. invasa*. Relatively higher expression levels of *EcDREB2* in leaf apex was expected, considering that *L. invasa* infestation primarily occurs in leaf tissues^12,81^. However, when the data was normalized with the least stable reference genes (Fig. 3), the expression pattern was modified and *EcDREB2* expression in leaf apex and stem showed no difference, thus confirming the influence of the reference genes used in the final result of the RT-qPCR gene expression analysis.

The results obtained in the study shows that the expression pattern of a target gene is closely associated with the reference genes used for data normalization. When employing reference genes with lower stability to calculate *EcDREB2* expression, a reduction in the relative expression level was observed, which can lead to erroneous interpretations and conclusions. These data highlight the relevance of carefully validating reference genes before applying them in gene expression studies.

## Conclusion

In the present study, for the first time, the analysis and selection of the best reference genes for gene expression normalization in *Eucalyptus* (*E. tereticornis* × *E. camaldulensis* hybrid) plants subjected to *B. bassiana* inoculation and *L. invasa* infestation has been conducted. *EcPTB*, *EcPP2A*-1, and *EcEUC12* were the best reference genes when all treatments were evaluated. The triplets *EcPTB*/*EcEUC12*/*EcUBP6*, *EcPP2A-1*/*EcEUC12*/*EcPTB*, *EcPP2A-1*/*Ecα-TUB*/*EcPTB*, *EcIDH*/*EcSAND*/*Ecα-TUB*, and *EcPP2A-1*/*EcUPL7*/*EcSAND* were the best reference genes for control plants, mother plants, plants infested with *L. invasa*, plants inoculated with *B. bassiana*, and plants inoculated with *B. bassiana* and infested with *L. invasa*, respectively. In relation to the worst reference genes, *EcPTB*, *EcPP2A-1*, and *EcEUC12* were defined as the least suitable genes when all treatments are evaluated. The data obtained highlight the importance of developing this type of study to increase the reliability of relative expression analyses. Furthermore, the reported findings can serve as a basis for the development of studies aimed at analyzing the relative expression of genes related to different metabolic pathways of interest, such as the defensive responses of eucalyptus plants inoculated with the entomopathogenic fungus *B. bassiana* and infested with the gall wasp *L. invasa*.

## Methods

### Experiment design

#### Study area

The experiment was carried out under greenhouse conditions at the Experimental Research Station of the Federal University of Tocantins – UFT, Gurupi, Tocantins, Brazil (11°43’ S and 49°04’ W, 284 m altitude). The region's climate is Aw type (tropical climate, with dry winter), with an average annual temperature and rainfall of 26.1 ºC and 1776.4 mm, respectively.

#### *Eucalyptus* seedling production

Rooted cuttings of the hybrid clone of *E. tereticornis* × *E. camaldulensis* were produced under greenhouse conditions. After 120 days, the cuttings were transplanted from tubes into 3.8-L pots containing Bioplant® (Bioplant, Ponte Nova, MG, Brazil) commercial substrate based on pine bark, carbonized rice husk, vermiculite, macronutrients, and micronutrients. After transplantation of the seedlings, plants were acclimatized in the greenhouse for 30 days and then taken to an area in full sun. The plant material used in this study complies with international, national and/or institutional guidelines.

#### B. bassiana inoculation

The fungus *B. bassiana* (strain PL 63) was obtained from the mycological collection of the Insect-Microorganism Symbioses Laboratory of UFT (Gurupi Campus). Plates from the collection were re-plated into new plates containing P.D.A. medium (Potato, Dextrose (Labsybth, Diadema, SP, Brazil), Agar (Labsybth, Diadema, SP, Brazil)), supplemented with amoxicillin (500 μg mL^−1^) (EMS Pharma Hortolândia, SP, Brazil), and maintained at B.O.D. (Biochemical Oxygen Demand) for a period of 12 days at 25 ºC ± 2 °C and a 12-h photoperiod.

After this period, plates were opened in a laminar flow chamber and, under aseptic conditions, spores were removed from the colonies. This step was performed by adding 10 ml of distilled water and Tween 80® 0.02% (v/v) (Labsybth, Diadema, SP, Brazil), previously autoclaved (121.0 °C for 15 min), with the spores being gently scraped with a sterilized spatula. These spores were transferred to a sterile beaker containing 100 ml of sterilized distilled water. This solution was stirred in an incubator with orbital shaking at 6 × g (RCF) for 10 min at room temperature. After this process, the suspension was filtered into an autoclaved (121.0 °C for 15 min) beaker through a double layer of sterilized gauze to retain the mycelium fragments and remains of the culture medium. An aliquot of this solution was placed in a Neubauer chamber to count spores using an optical microscope. Then, the concentration of the solution was adjusted to 10^8^ spores/ml with distilled water containing 0.02% (v/v) Tween 80®. The inoculation with this solution was performed immediately after its preparation. For control plants, an autoclaved (121.0 °C for 15 min) solution of distilled water solution containing 0.02% (v/v) Tween 80® was used. From the same solution, a 15 ml aliquot was used to perform the viability test with the aid of the Neubauer chamber. After a period of 48 h, the viability test was carried out, where the conidia evaluated displayed a viability of 80%. The adaxial epidermis of the fourth, fifth and sixth fully expanded leaves from each plant were slightly injured with a soft sponge. Spraying was carried out, especially on injured leaves. After spraying, six branches from the upper third of each plant were covered with transparent plastic bags for 36 h^82^.

#### L. invasa breeding and infestation

The *L. invasa* individuals used in this work were obtained from a breeding cage (2.8 m × 5.2 m × 3.0 m—height × length × width, and branches with galls close to emergence were cut from eucalyptus plants (hybrid *E. tereticornis* × *E. camaldulensis*) in the breeding cage. After cutting the branches, they were immediately taken to the laboratory and placed in a beaker with distilled water inside a bench cage covered with organza. A container with a solution of water and honey (3:2 ratio) was also placed inside the cage to feed the wasp individuals that emerged from the galls. The cage was kept in the laboratory, at room temperature, for a period of 24 h. After this period, the individuals that emerged were collected with manual suckers and placed in microcentrifuge tubes.

At 45 days after inoculation of *B. bassiana* in eucalyptus plants, all plants (inoculated and non-inoculated), except control and mother plants, were infested with *L. invasa*. For this, six branches of each plant were wrapped in an organza bag containing a closed 2.0 ml microcentrifuge tube with two individuals of *L. invasa*^10^. Then, the microtubes were opened so that the wasps could come out to oviposit. After 48 h of infestation, the bags and wasps were removed, and after 48 h from this, samples were collected.

#### Plant material and treatments

Samples consisted of roots, stems, fully expanded leaves, and leaf apices from mother plants maintained under field conditions and the plants produced as previously described. Each of these materials were collected from plants of the following treatments: mother plants, control plants (without inoculation and without infestation), plants infested with *L. invasa*, seedlings inoculated with *B. bassiana*, and plants inoculated with *B. bassiana* and also infested with *L. invasa*. Three biological replicates were used for each sample type, with each biological replicate consisting of four plants. Samples were collected and immediately frozen in liquid nitrogen and subsequently stored at − 80 °C until RNA extraction. Molecular analyses were carried out at the Molecular Analysis Laboratory (LAM) of the Federal University of Tocantins (UFT), Palmas campus.

#### RNA extraction

RNA extraction was performed using the CTAB (cetyltrimethylammonium bromide) method^83^ modified by Gonçalves et al.^84^ and with minor alterations (Supplementary S2). After extraction, the RNA quantity and purity (A_260/A280_ and A_260/A230_ ratios) were determined through a spectrophotometer (Nanodrop® One Spectrophotometer, Thermo Fisher Scientific, Wilmington, DE), while RNA integrity was verified using the agarose gel (0.8%).

#### DNase treatment and cDNA synthesis

RNA samples (5 μg) were treated with DNase I, using the Turbo DNA-free kit (Applied Biosystems, Thermo Fisher Scientific, Vilnius, Lithuania) and following its instructions, to eliminate residual DNA contamination. Subsequently, RNA was evaluated for its quantity and purity (A_260_/A_280_ and A_260_/A_230_ ratios) through spectrophotometry (Nanodrop® One Spectrophotometer) and its integrity was analyzed through agarose gels (0.8%). cDNA was synthesized from 1.0 μg of RNA using the High-Capacity cDNA Reverse Transcription kit (Invitrogen, Thermo Fisher Scientific, Vilnius, Lithuania) and following the manufacturer's protocol. cDNA samples were then stored at − 20 °C.

#### Identification and selection of target and reference genes

The reference genes analyzed in this study were chosen from a search for eucalyptus reference gene studies on the Web of Science database (www.webofknowledge.com), using the following keywords: *housekeeping gene*, *endogenous gene*, *reference gene*, and *Eucalyptus*. The Boolean interpolator “and” was used. From this search, the selection of the reference genes followed the recommendation criteria of the article in which they were analyzed, that is, the genes that had the best results (greater expression stability in the sample set used) were prioritized. Following this approach, 12 different genes were selected: *PP2A-1*, *SAND*, *UPL7*, *PTB*^38^; *Actin-2*, *18srRNA*, *IDH*^34^; *α-TUB*, *UBC*, *EF-1α*^37^; *EUC12*, and *H2B*^36^.

The selection of the target gene used to validate the selected reference genes was based on the gene expression data generated by the tool (https://plantgenie.org/) described by Sundell et al.^42^. For this, the PFAM number (PF00847)^85^ corresponding to the AP2/EREBP superfamily was used to search on the available *E. grandis* database of the tool, all gene sequences referring to this superfamily. After identifying the sequences, they were selected and used to generate a gene expression graph using the ExHeatmap option. After analyzing the graph, the gene *DREB2* (Eucgr.F02440.1) showed a high expression level in *E. grandis* tissues subjected to *L. invasa* infestation, being selected for validation analysis.

#### Gene sequence identification

The reference and target gene sequences were obtained using the BLAST tool (Basic Local Alignment Search)^86^ through the comparison of their nucleotide sequences from *E. grandis*, obtained on the Phytozome database (https://phytozome-next.jgi.doe.gov/), with the *Eucalyptus camaldulensis* Genome Database (https://www.kazusa.or.jp/eucaly/index.html).

#### Primer design

RT-qPCR primers for RT-qPCR (Table 6) were designed by using the reference and target gene sequences obtained from the *Eucalyptus camaldulensis* Genome Database and the OligoPerfect program (apps.thermofisher.com/apps/oligoperfect/). Quality assessment of the designed primers was evaluated by the OligoAnalyzer tool (http://www.idtdna.com/calc/analyzer), and the melting curves of all genes analyzed here are described in Supplementary S3. Corrections of primer efficiency differences were performed by applying the mathematical model proposed by Pfaffl^49^.

**Table 6:** RT-qPCR primer sequences, gene name, accession number, melting temperature (Tm), amplicon size, correlation coefficient (R^2^), and amplification efficiency (E%) of the reference and target genes analyzed.

Gene name	Accession number	Primer sequence (5′–3′)	Tm (°C)	Amplicon (bp)	R^2^	E (%)
*EcPP2A-1*	EcC035070.20	Fw: GGAGATTGGAGAGAACATGGACC Rv: GGAGTCTTGCGTGTGGTGTC	62.5 60.7	87	0.99	100.0
*EcEF-1α*	EcC054749.90	Fw: TCGGGCTTTGAGGGTGACAA Rv: TCTTGGGCTCTAGGATCAGGTC	61.6 62.8	103	1.00	97.7
*EcPTB*	EcC038236.10	Fw: TTTCTGCTTTTGGTCCTGTGC Rv: GCAGCAGTCGTTATGTCAGGG	60.8 62.5	94	0.99	96.8
*EcACT*	EcS556981.10	Fw: GCTGGATTCGCAGGTGATGATG Rv: CCTTCTGACCCATTCCGACCA	62.8 64.8	97	1.00	100.0
*EcIDH*	EcC000103.10	Fw: AGCGAGGGAGGTTATGTGTGG Rv: ATGTCATCAAGCCAAGCGATCC	63.7 61.9	97	0.98	96.2
*Ecα-TUB*	EcC009196.10	Fw: GGGGATTCAGGTCGGCAACT Rv: TGAGGTGTCACTGGGCATCG	62.3 61.7	88	1.00	95.1
*EcUBC2*	EcS510545.10	Fw: TGCTCTTATCAAGGGACCGTCG Rv: GAAACGAACTTGAGGCGGCT	63.7 60.1	106	0.99	100.0
*EcEUC12*	EcC054911.20	Fw: TACCCTGTGAGAGTGCTGCC Rv: GCACATCTCCCGCTCATCCT	60.7 61.2	90	0.99	97.4
*EcH2B*	EcC049406.40	Fw: CCTGCTCGATTCCTGATGGC Rv: CACCGCCGACTTCTTCTCCT	60.3 61.6	81	0.99	100.0
*EcSAND*	EcC020159.20	Fw: TGAGGAAGTGAGGAGTGACGC Rv: CATCATCCTCATCAACGTGCCG	63.8 63.1	80	0.99	99.3
*EcUPL7*	EcC023921.10	Fw: ACTGAACCCTGTGGCTGGAT Rv: GCGATCAGAGGCACCATGAAA	60.4 62.2	118	0.97	83.1
*EcDREB2*	EcC047161.20	Fw: GAGGTGACGGAGGTGAAGGG Rv: GCAACGAAACGCACTCCCAC	63.0 61.7	118	1.00	87.4

#### RT-qPCR analysis

RT-qPCR analyses were carried out on an ABI PRISM 7500 Real-Time PCR thermocycler (Applied Biosystems, Thermo Fisher Scientific, Singapore), using the PowerUp™ SYBR™ Green Master Mix (Applied Biosystems, Thermo Fisher Scientific, Vilnius, Lithuania). Reactions were performed in 10 μL final volume: 1.0 μL of cDNA (diluted 1:5), 0.2 μL of each primer at 10 μM, and 5.0 μL of PowerUp™ SYBR™ Green Master Mix, and 3.6 μL of RNase-DNase-free water. Three biological replicates were used, and reactions were run in triplicates as technical repetitions. Amplification reactions were carried out with the following conditions: 2 min at 50 °C, 2 min at 95 °C, followed by 40 cycles of 15 s at 95 °C and 1 min at 60 °C. To confirm the specificity of the primers, melting curves were generated after 40 amplification cycles for each primer pair by raising the temperature from 60 to 95 °C, with 1 °C increase in temperature every 5 s. Cq was determined by the number of cycles in which the fluorescence generated within a reaction crosses the threshold line.

#### Expression stability and validation of candidate reference genes

The algorithms geNorm^59^, NormFinder^62^, BestKeeper^63^, and Delta-Ct^64^ were used to calculate the stability values. The algorithms generate a classification based on the stability value of each evaluated gene. Subsequently, the data generated by the algorithms were analyzed by RefFinder, which integrates the four algorithms and provides a general classification of all candidate reference gene tested^65,66^. The following sample sets were used by the RefFinder tool (www.ciidirsinaloa.com.mx/RefFinder-master/) to evaluate the stability of the evaluated genes: root, stem, leaf and leaf apex in all treatments; and root, stem, leaf, and leaf apex in each treatment. Box plots were plotted to illustrate the expression levels and variations of the tested genes by the SigmaPlot program.

Reference gene validation was performed through expression analyzes of the target gene, *EcDREB2.* The mathematical model proposed by Pfaffl^49^, which is based in primer amplification efficiencies, was used to calculate the relative expression values. Expression data was normalized by using more than one reference gene, in accordance with Bustin et al.^46^ *EcDREB2* expression pattern was normalized with the most (*EcPP2A-1*, *EcULP7* and *EcSAND*) and least (*EcH2B*, *EcEF1-α* e *EcACT*) stable reference genes, according to the RefFinder algorithm, and analyzed in stem and leaf apex from eucalyptus plants submitted to *L. invasa* infestation and *B. Bassiana* inoculation. Relative expression graphs were plotted using the SigmaPlot program (version 12.0, Systat Software Inc, San Jose, CA, USA).

### Supplementary Information

Supplementary Information 1.

Supplementary Information 2.

Supplementary Information 3.

## Data Availability

All data generated and analyzed for this study are included in this published article and its Supplementary Information files. All programs used to analyze the data are publicly available.
