# Supplementary material for: Reference genes for Eucalyptus spp. under Beauveria bassiana inoculation and subsequently infestation by the galling wasp Leptocybe invasa
Source: Sci Rep. 2024 Jan 31;14:2556. doi: 10.1038/s41598-024-52948-x (PMC10830493; doi:10.1038/s41598-024-52948-x)
Supplement: Supplementary file 1 — Supplementary Information 1. [file 41598_2024_52948_MOESM1_ESM.pdf]

Reference genes for *Eucalyptus* spp. under *Beauveria bassiana* inoculation and subsequently infestation by the galling wasp *Leptocybe invasa*

Matheus Martins Daude[1,2]; Solange Aparecida Ságio[1,3]; Jovielly Neves Rodrigues[4], Nívea Maria Pereira Lima[5], André Almeida Lima[1]; Maíra Ignacio Sarmento[4]; Renato Almeida Sarmento[2,4]; Horllys Gomes Barreto[*1,2,3].

[1]Laboratory of Molecular Analysis (LAM), Life Sciences Department, Faculty of Medicine, Federal University of Tocantins, Palmas, TO, Brazil.

[2] Graduate Program in Biotechnology and Biodiverty, Rede Bionorte, Federal University of Tocantins, Palmas, TO, Brazil.

[3] Graduate Program in Digital Agroenergy, Federal University of Tocantins, Palmas, TO, Brazil.

[4]Graduate Program in Forest and Environmental Sciences, Federal University of Tocantins, Palmas, TO, Brazil.

[5]Agronomy Undergraduate course, Federal University of Tocantins, Palmas, TO, Brazil

*Correspondence:

E-mail: horllys@uft.edu.br

SUPPLEMENTARY S1

Table 1 Cycle of quantification (Cq) for every samples and genes of the eucalyptus mother plants. Each sample was composed by three biological replicates and one of them was run in technical triplicates. The letters S, L, and LA refer to Stem, Leaves, and Leaf Apex, respectively. The numbers right after the letters representing the sample type refer to the number of the biological replicate. *Ec* = *Eucalyptus camaldulensis.*

SAMPLE	*EcEF1-α*	*EcPP2A-1*	*EcACT*	*EcTUB*	*EcPTB*	*EcUBP6*	*EcH2B*	*EcEUC12*	*EcIDH*	*EcSAND*	*EcUPL7*
S1	24.56	23.78	17.31	21.49	23.92	21.74	20.43	22.93	19.61	22.71	24.94

S1	24.53	23.59	17.36	21.35	23.65	21.50	20.20	22.75	19.43	22.64	24.87
S1	24.65	23.41	17.33	21.34	23.61	21.53	20.18	22.67	19.79	22.78	24.61
S2	23.16	22.53	15.99	20.66	23.17	20.59	19.48	21.57	19.15	22.07	24.41
S2	23.04	22.28	16.19	20.49	22.97	20.48	19.22	21.51	18.69	22.04	24.30
S2	23.06	22.25	16.64	20.48	22.93	20.41	19.26	21.45	18.88	22.10	24.32
S3	23.43	22.62	16.22	20.85	23.49	20.67	19.44	21.90	19.25	22.41	24.71
S3	23.32	22.48	16.24	20.58	23.16	20.54	19.39	21.78	18.98	22.62	24.85
S3	23.20	22.31	16.65	20.43	23.04	20.54	19.23	21.78	18.99	22.32	24.79
L1	28.77	25.98	22.52	24.26	26.15	22.48	23.43	25.58	21.68	24.51	27.66
L1	28.49	25.77	22.58	24.28	25.91	22.32	23.33	25.62	21.59	24.42	27.75
L1	28.38	25.80	22.50	23.87	25.85	22.37	23.39	25.28	21.56	24.60	27.78
L2	28.10	25.73	21.86	23.64	25.67	22.15	22.78	24.97	21.03	24.81	27.19
L2	27.73	25.45	21.84	23.61	25.61	22.22	22.87	25.06	20.78	24.85	27.09
L2	27.98	25.23	21.78	23.49	25.63	22.63	22.78	24.93	20.88	24.78	27.18
L3	26.92	24.77	20.19	23.24	25.41	21.76	22.48	24.63	20.73	23.72	26.75
L3	26.94	24.68	20.21	23.14	25.14	21.68	22.30	24.47	20.33	23.72	26.75
L3	27.01	24.64	20.47	23.04	25.05	21.69	22.25	24.29	20.47	23.72	26.68

LA1	24.03	23.13	17.43	21.29	24.16	21.82	17.56	23.32	20.60	22.52	26.24
LA1	24.04	23.14	17.22	21.11	23.90	21.58	17.16	23.02	20.41	22.50	26.04
LA1	23.98	23.00	17.41	20.98	23.83	21.49	17.97	22.91	20.32	22.54	26.03
LA2	24.07	23.31	18.74	20.98	23.98	21.54	18.17	23.11	20.04	23.48	25.67
LA2	24.18	23.27	18.55	20.71	23.79	21.54	19.97	22.93	19.79	23.17	25.51
LA2	24.12	23.20	18.47	20.66	23.83	21.44	17.89	22.78	19.63	22.99	25.36
LA3	24.34	23.65	18.66	21.24	23.90	21.62	18.31	23.24	20.31	23.37	25.91
LA3	24.37	23.62	18.53	21.11	23.84	21.60	18.32	23.03	20.17	23.22	25.60
LA3	24.29	23.60	18.48	20.87	23.70	21.50	18.21	22.87	20.09	23.25	25.66

Table 2 Cycle of quantification (Cq) for every samples and genes of the eucalyptus control plants. Each sample was composed by three biological replicates and one of them was run in technical triplicates. The letters R, S, L, and LA refer to Roots, Stem, Leaves, and Leaf Apex, respectively. The numbers right after the letters representing the sample type refer to the number of the biological replicate. *Ec* = *Eucalyptus camaldulensis.*

**SAMPLE**	***EcEF1-α***	***EcPP2A-1***	***EcACT***	***EcTUB***	***EcPTB***	***EcUBP6***	***EcH2B***	***EcEUC12***	***EcIDH***	***EcSAND***	***EcUPL7***
R1	23.63	23.68	18.55	22.27	23.87	21.31	19.49	22.59	18.69	23.15	25.82
R1	23.63	23.63	18.40	22.11	23.96	21.18	19.45	22.53	18.62	23.01	25.53
R1	23.67	23.49	18.28	22.00	23.67	21.00	19.53	22.44	18.65	23.09	25.54

R2	23.82	23.79	18.30	22.17	23.71	20.92	20.37	22.43	19.40	22.26	25.35
R2	23.95	23.73	18.20	22.14	23.68	20.76	20.27	22.36	18.99	21.91	25.43
R2	23.83	23.68	18.07	21.90	23.61	20.76	20.32	22.18	19.39	22.15	25.27
R3	23.67	23.51	18.33	21.74	23.96	21.16	20.52	22.71	20.08	21.65	25.73
R3	23.52	23.48	18.15	21.57	23.90	20.93	20.34	22.41	19.79	21.58	25.46
R3	23.51	23.48	18.40	21.51	24.09	20.88	20.27	22.35	19.80	21.64	25.40
S1	23.77	22.92	17.46	20.73	23.29	20.36	19.62	21.76	18.96	22.17	24.84
S1	23.66	22.75	17.24	20.69	23.08	20.10	19.41	21.59	18.74	22.09	24.77
S1	23.57	22.73	17.19	20.68	22.97	20.21	19.29	21.59	19.16	21.88	24.91
S2	23.28	22.44	17.41	20.75	23.28	20.30	19.26	21.74	18.95	21.85	24.78
S2	23.36	22.32	17.41	20.82	23.23	20.09	19.33	21.62	19.02	21.75	24.66
S2	23.39	22.33	17.46	20.78	23.24	20.11	19.31	21.64	19.30	21.93	24.42
S3	23.39	22.66	17.50	20.96	23.50	20.61	19.28	21.80	19.28	21.92	24.76
S3	23.41	22.47	17.40	20.86	23.30	20.34	19.25	21.80	19.22	21.81	24.68
S3	23.41	22.39	17.38	20.81	23.34	20.34	19.24	21.63	19.17	21.76	24.59
L1	26.41	24.61	20.95	22.88	24.98	21.17	20.68	23.58	21.66	23.67	25.75
L1	26.35	24.33	20.93	22.97	24.92	20.97	20.67	23.90	21.52	23.62	25.54

L1	26.34	24.36	20.81	22.80	24.87	20.93	20.53	23.45	21.59	23.25	25.48
L2	25.49	24.04	19.99	22.51	24.36	20.97	20.21	22.75	21.70	23.45	25.21
L2	25.59	23.93	19.82	22.09	24.25	20.81	20.19	22.79	21.64	23.43	25.35
L2	25.64	23.93	19.80	21.96	24.29	20.77	19.96	22.70	21.77	23.27	25.20
L3	25.92	23.80	19.81	22.00	24.31	20.69	19.13	22.99	20.86	23.65	25.54
L3	25.84	23.85	19.75	22.02	24.11	20.75	19.13	22.80	20.10	23.56	25.41
L3	25.93	23.88	19.76	22.21	24.13	20.67	18.93	22.87	20.18	23.58	25.42
LA1	26.02	24.33	18.96	22.53	24.76	22.09	19.35	23.24	20.69	24.35	26.71
LA1	26.18	24.28	18.64	22.48	24.60	21.99	19.23	23.26	20.69	24.32	26.87
LA1	25.79	24.11	18.80	22.60	24.53	21.95	18.96	23.03	20.70	24.31	26.75
LA2	27.48	25.33	20.13	23.80	25.61	22.39	21.64	23.81	20.84	24.65	27.12
LA2	27.36	25.25	19.77	23.61	25.32	22.44	21.25	23.75	20.76	24.49	26.98
LA2	27.38	25.08	19.95	23.72	25.47	22.47	21.51	23.70	20.93	24.39	27.05
LA3	25.19	24.21	19.00	21.91	24.34	21.71	18.90	23.03	20.95	22.90	26.16
LA3	25.09	24.00	18.88	21.91	24.31	21.66	18.76	22.85	20.81	23.27	26.22
LA3	25.08	24.08	18.85	21.83	24.28	21.62	18.35	22.86	20.67	22.96	26.27

Table 3 Cycle of quantification (Cq) for every samples and genes of the eucalyptus plants infested with the *L. invasa* wasp. Each sample was composed by three biological replicates and one of them was run in technical triplicates. The letters S, L, and LA refer to Stem, Leaves, and Leaf Apex, respectively. The numbers right after the letters representing the sample type refer to the number of the biological replicate. *Ec* = *Eucalyptus camaldulensis.*

SAMPLE	*EcEF1-α*	*EcPP2A-1*	*EcACT*	*EcTUB*	*EcPTB*	*EcUBP6*	*EcH2B*	*EcEUC12*	*EcIDH*	*EcSAND*	*EcUPL7*
S1	23.86	23.22	17.46	21.15	23.78	20.71	19.24	21.93	19.16	22.85	25.12
S1	23.86	23.22	17.65	21.00	23.57	20.57	19.19	21.95	18.82	22.73	25.14
S1	23.87	23.22	17.85	20.95	23.62	20.50	19.13	21.90	19.04	22.64	24.97
S2	23.80	23.36	17.81	21.06	23.73	20.95	18.64	22.00	19.68	22.42	24.30
S2	23.85	23.15	17.20	21.07	23.74	21.25	18.42	22.12	19.54	22.48	24.86
S2	23.91	23.20	17.50	20.94	23.73	20.79	18.20	22.31	19.83	22.36	24.68
S3	23.90	23.35	17.93	21.28	23.99	21.00	18.76	22.45	19.98	22.81	24.75
S3	23.96	23.12	17.26	21.16	23.90	21.11	18.71	22.37	19.71	22.73	24.69
S3	24.04	23.12	17.59	21.12	23.90	20.81	18.66	22.38	19.89	22.70	24.72
L1	24.74	23.22	19.28	21.44	23.70	20.54	19.60	22.55	18.55	21.92	24.49
L1	24.68	23.19	18.99	21.40	23.61	20.51	19.56	22.55	18.44	21.75	24.43
L1	24.58	23.11	19.13	21.41	23.99	20.52	19.55	22.60	18.66	21.97	24.34
L2	25.70	24.14	20.22	22.02	24.09	21.03	19.54	23.20	19.53	23.23	25.59

L2	25.64	23.97	20.04	22.02	24.25	20.85	19.77	23.13	19.48	23.27	25.51
L2	25.75	24.04	20.30	22.21	24.51	20.79	19.71	23.26	19.59	23.49	25.55
L3	25.45	23.82	19.98	21.86	23.88	20.81	19.32	23.28	19.50	22.63	25.68
L3	25.41	23.77	19.92	21.81	24.05	20.83	19.50	23.34	19.34	22.68	25.43
L3	25.45	23.48	20.07	21.97	24.29	20.83	19.19	23.33	19.66	22.74	25.18
LA1	24.93	23.34	18.57	21.40	23.75	21.52	17.88	22.44	20.89	22.97	24.85
LA1	24.23	23.25	18.44	21.44	23.86	21.56	17.96	23.43	20.63	22.92	24.81
LA1	24.29	23.19	18.48	21.43	24.02	21.47	17.66	22.51	20.81	22.81	24.90
LA2	24.32	23.34	18.65	21.22	23.86	21.36	19.12	22.38	19.71	22.61	24.51
LA2	24.27	23.32	18.47	21.22	23.71	21.40	18.76	22.22	19.50	22.60	24.51
LA2	24.29	23.22	18.47	21.16	23.94	21.43	18.72	22.19	19.71	22.42	24.51
LA3	24.47	23.34	18.78	21.56	24.02	21.66	18.80	22.57	19.52	23.16	25.85
LA3	24.48	23.48	18.54	21.51	23.84	21.44	18.35	22.51	19.43	22.96	25.71
LA3	24.50	23.48	18.60	21.48	24.36	21.54	18.21	22.35	19.62	22.99	25.76

Table 4 Cycle of quantification (Cq) for every samples and genes of the eucalyptus plants inoculated with the *B. bassiana* fungus. Each sample was composed by three biological replicates and one of them was run in technical triplicates. The letters S, L, and LA refer to Stem, Leaves, and Leaf Apex, respectively. The numbers right after the letters representing the sample type refer to the number of the biological replicate. *Ec* = *Eucalyptus camaldulensis.*

SAMPLE	*EcEF1-α*	*EcPP2A-1*	*EcACT*	*EcTUB*	*EcPTB*	*EcUBP6*	*EcH2B*	*EcEUC12*	*EcIDH*	*EcSAND*	*EcUPL7*
S1	24.12	23.37	18.19	21.35	24.55	21.17	19.39	22.99	19.94	22.85	24.70
S1	24.05	23.34	17.90	21.18	24.13	20.99	19.17	22.76	19.81	22.62	24.57
S1	23.99	23.33	17.93	21.23	24.17	20.90	19.14	22.66	19.78	22.50	24.58
S2	23.83	22.90	17.86	21.16	24.08	20.86	19.05	22.63	19.53	22.52	24.54
S2	23.64	22.86	17.53	20.97	23.86	20.61	18.97	22.44	19.36	22.22	24.54
S2	23.73	22.85	17.56	20.88	23.83	20.52	18.88	22.28	19.32	22.28	24.71
S3	24.08	23.33	18.14	21.41	24.22	20.84	19.17	22.68	19.80	22.73	24.87
S3	23.95	23.12	18.13	21.32	24.12	20.75	19.07	22.56	19.75	22.61	24.86
S3	23.92	23.13	17.89	21.21	23.98	20.67	18.93	23.14	19.55	22.59	25.14
L1	24.67	23.37	19.42	21.57	24.36	20.73	19.56	22.52	20.26	22.44	24.65
L1	24.56	23.42	19.28	21.53	24.28	20.54	19.52	22.59	20.11	22.43	24.76
L1	24.55	23.41	19.24	21.41	24.29	20.55	19.48	22.57	20.00	22.41	24.80
L2	24.97	23.74	19.42	21.85	24.13	20.68	19.83	22.56	20.15	22.72	24.72

L2	24.81	23.54	19.36	21.73	23.93	20.50	19.67	22.63	20.15	22.58	24.74
L2	24.78	23.43	19.31	21.69	23.89	20.57	19.76	22.65	20.05	22.57	24.85
L3	25.89	24.78	20.61	22.79	25.03	21.34	21.09	23.48	21.05	23.46	25.82
L3	25.91	24.62	20.45	22.75	24.92	21.18	20.81	23.57	20.85	23.53	25.75
L3	25.79	24.54	20.42	22.65	24.70	21.08	20.92	23.51	20.82	23.36	25.75
LA1	27.67	25.84	21.68	24.34	26.36	22.99	21.11	24.26	22.81	24.95	27.43
LA1	27.45	25.88	21.22	24.00	26.14	22.73	21.95	24.35	22.64	24.75	27.32
LA1	27.52	25.70	21.26	24.01	26.10	22.72	21.75	24.19	22.62	24.75	27.41
LA2	28.96	26.36	22.45	25.26	26.85	23.84	23.03	25.25	23.62	25.73	28.19
LA2	29.50	26.50	22.34	24.91	26.96	23.53	22.58	25.06	23.54	25.70	27.88
LA2	28.31	26.74	22.24	24.95	26.74	23.49	22.59	25.13	23.41	25.55	28.17
LA3	30.31	27.34	24.39	26.95	27.97	24.63	24.45	26.80	25.01	27.21	29.70
LA3	30.66	27.19	24.35	26.86	27.92	24.43	24.28	26.41	24.82	27.23	29.62
LA3	30.13	27.66	24.21	26.65	27.88	24.47	24.26	26.21	24.75	27.10	29.49

Table 5 Cycle of quantification (Cq) for every samples and genes of the eucalyptus plants infested with the *L. invasa* wasp and inoculated with the *B. bassiana* fungus. Each sample was composed by three biological replicates and one of them was run in technical triplicates. The letters S, L, and LA refer to Stem, Leaves, and Leaf Apex, respectively. The numbers right after the letters representing the sample type refer to the number of the biological replicate. *Ec* = *Eucalyptus camaldulensis.*

SAMPLE	*EcEF1-α*	*EcPP2A-1*	*EcACT*	*EcTUB*	*EcPTB*	*EcUBC6*	*EcH2B*	*EcEUC12*	*EcIDH*	*EcSAND*	*EcUPL7*
S1	29.57	27.10	22.55	25.66	27.07	23.33	23.96	25.76	22.91	26.19	29.08
S1	29.80	27.01	22.52	25.62	26.89	23.27	23.88	25.81	22.81	26.03	28.96
S1	29.68	27.15	22.31	25.44	26.87	23.15	23.86	25.77	22.70	25.79	28.75
S2	29.62	27.25	22.65	25.52	26.98	23.23	23.94	25.52	22.95	26.03	28.99
S2	29.55	26.96	22.49	25.37	26.74	22.94	23.85	25.57	22.78	25.66	29.15
S2	29.43	27.28	22.41	25.32	26.88	22.84	23.87	25.65	22.61	25.76	28.71
S3	26.18	24.58	19.11	21.87	23.23	21.53	20.81	23.41	20.18	23.71	26.29
S3	26.04	24.41	19.21	21.81	23.83	21.43	20.68	23.21	20.10	23.48	26.18
S3	25.96	24.88	19.01	21.73	23.53	21.30	20.69	23.21	19.93	23.42	25.96
L1	25.75	24.05	19.91	21.53	24.66	21.07	20.72	23.44	20.21	23.41	25.96
L1	25.82	24.00	19.68	21.47	24.42	20.85	20.50	23.19	19.86	23.28	25.74
L1	25.73	23.98	19.74	21.45	24.41	20.73	20.53	23.21	20.06	23.08	25.81

L2	26.73	25.75	21.69	24.03	26.18	20.08	21.75	24.84	21.58	24.69	27.28
L2	26.82	25.78	21.62	24.00	26.00	22.09	21.55	24.76	21.46	24.74	26.75
L2	26.87	25.83	21.65	24.00	25.82	21.94	21.49	24.64	21.55	24.71	27.08
L3	28.32	26.65	22.62	24.34	27.39	23.30	22.92	25.97	22.45	25.62	28.50
L3	28.40	26.66	22.62	24.32	26.98	23.29	22.82	25.69	22.40	25.53	28.47
L3	28.60	26.67	22.50	24.30	26.98	23.30	23.02	25.84	22.43	25.65	28.36
LA1	24.95	23.85	18.79	21.90	24.21	21.16	19.21	22.77	20.43	23.07	25.54
LA1	25.05	23.73	18.82	21.73	24.22	21.57	19.19	22.68	20.44	22.95	25.52
LA1	24.99	23.75	18.70	21.80	24.14	21.48	19.34	22.54	20.47	22.91	25.49
LA2	25.06	24.04	18.77	21.81	24.37	21.76	19.04	22.81	20.83	23.50	25.65
LA2	25.07	23.85	18.78	21.77	24.10	21.65	18.89	22.64	20.66	23.44	25.52
LA2	25.01	23.89	18.76	21.74	24.15	21.55	18.92	22.63	20.75	23.23	25.66
LA3	25.48	24.53	19.31	22.00	24.41	21.94	19.72	23.20	20.67	23.55	25.91
LA3	25.40	24.41	19.28	22.06	24.34	21.80	19.57	23.14	20.58	23.43	26.14
LA3	25.44	24.36	19.25	22.08	24.25	21.90	19.58	23.13	20.41	23.40	25.81
