# Supplementary material for: Reference genes for Eucalyptus spp. under Beauveria bassiana inoculation and subsequently infestation by the galling wasp Leptocybe invasa
Source: Sci Rep. 2024 Jan 31;14:2556. doi: 10.1038/s41598-024-52948-x (PMC10830493; doi:10.1038/s41598-024-52948-x)
Supplement: Supplementary file 2 — Supplementary Information 2. [file 41598_2024_52948_MOESM2_ESM.pdf]

Reference genes for *Eucalyptus* spp. under *Beauveria bassiana* inoculation and subsequently infestation by the galling wasp *Leptocybe invasa*

Matheus Martins Daude[1,2]; Solange Aparecida Ságio[1,3]; Jovielly Neves Rodrigues[4], Nívea Maria Pereira Lima[5], André Almeida Lima[1]; Maíra Ignacio Sarmento[4]; Renato Almeida Sarmento[2,4]; Horllys Gomes Barreto[*1,2,3].

[1]Laboratory of Molecular Analysis (LAM), Life Sciences Department, Faculty of Medicine, Federal University of Tocantins, Palmas, TO, Brazil.

[2] Graduate Program in Biotechnology and Biodiverty, Rede Bionorte, Federal University of Tocantins, Palmas, TO, Brazil.

[3] Graduate Program in Digital Agroenergy, Federal University of Tocantins, Palmas, TO, Brazil.

[4]Graduate Program in Forest and Environmental Sciences, Federal University of Tocantins, Palmas, TO, Brazil.

[5]Agronomy Undergraduate course, Federal University of Tocantins, Palmas, TO, Brazil

*Correspondence:
E-mail: horllys@uft.edu.br

Supplementary S2

Detailed description of the RNA extraction protocol used for eucalyptus tissues.

1° step: add 525 μL of extraction buffer [2 % (p/v) CTAB (cetyltrimethylammonium bromide), PVP 2 % (p/v), Tris-HCL (100 mM), EDTA (25 mM), NaCl (20 mM)], 525 μL de TES [TRIS-HCL (10 Mm) pH 7,5, EDTA (10 mM) SDS (0,5 %)], and 250 μL of β-mercaptoetanol for 100 ng of plant tissue;

2° step: mix the samples (vortex) for 45 s and incubate them for one hour (mix samples every 10 min) at 65 °C;

3° step: add 1,7 mL of chloroform and mix (vortex) samples for 45 s;

4° step: centrifuge samples for 10 min at 4 °C and 14,000 RPM;

5° step: transfer 800 μL of the top aqueous phase to a new 2 mL centrifuge tube (2 mL). Add 1 mL chloroform and mix (vortex) samples for 45 s;

6° step: centrifuge samples for 10 min at 4 °C and 14,000 RPM;

Thereafter, for leaf apex:

7° step: transfer 400 µL of the top aqueous phase to a new 1.5 mL centrifuge tube, add 400 µL chloroform, and mix (vortex) samples for 45 s;

8° step: centrifuge samples for 10 min at 4 °C and 14,000 RPM;

9° step: transfer 150 µL of the supernatant to a new 1.5 mL centrifuge tube. Add 150 µL of isopropyl alcohol and mix samples by inversion (15 times).

Thereafter, for roots, stem and leaves:

7° step: transfer 300 µL of the top aqueous phase to a new 1.5 mL centrifuge tube. Add 300 µL of isopropyl alcohol and mix samples by inversion (15 times);

After performing these specific steps for each plant tissue, the following steps were common for every tissue:

10° or 8° step: incubate samples for one hour at -20 °C;

11° or 9° step: centrifuge samples for 30 min at 4 °C and 14,000 RPM;

12° or 10° step: discard the supernatant and wash the pellet by adding 800 µL of 75 % ethanol;

13° or 11° step: centrifuge samples for 8 min at 4 °C and 14,000 RPM;

14° or 12° step: discard the ethanol and let the pellets dry (centrifuge tubes opened) for 5 min at 37 °C;

15° or 13° step: resuspend the pellets with 20 µL of RNase-free water.
