# Supplementary material for: Reference genes for Eucalyptus spp. under Beauveria bassiana inoculation and subsequently infestation by the galling wasp Leptocybe invasa
Source: Sci Rep. 2024 Jan 31;14:2556. doi: 10.1038/s41598-024-52948-x (PMC10830493; doi:10.1038/s41598-024-52948-x)

Reference genes for *Eucalyptus* spp. under *Beauveria bassiana* inoculation and subsequently infestation by the galling wasp *Leptocybe invasa*

Matheus Martins Daude[1,2]; Solange Aparecida Ságio[1,3]; Jovielly Neves Rodrigues[4], Nívea Maria Pereira Lima[5], André Almeida Lima[1]; Maíra Ignacio Sarmento[4]; Renato Almeida Sarmento[2,4]; Horllys Gomes Barreto[*1,2,3].

[1]Laboratory of Molecular Analysis (LAM), Life Sciences Department, Faculty of Medicine, Federal University of Tocantins, Palmas, TO, Brazil.

[2] Graduate Program in Biotechnology and Biodiverty, Rede Bionorte, Federal University of Tocantins, Palmas, TO, Brazil.

[3] Graduate Program in Digital Agroenergy, Federal University of Tocantins, Palmas, TO, Brazil.

[4]Graduate Program in Forest and Environmental Sciences, Federal University of Tocantins, Palmas, TO, Brazil.

[5]Agronomy Undergraduate course, Federal University of Tocantins, Palmas, TO, Brazil

SUPPLEMENTARY S3

Figure 1 *Melting* curves of the candidate reference genes analyzed.

EcPP2A-1

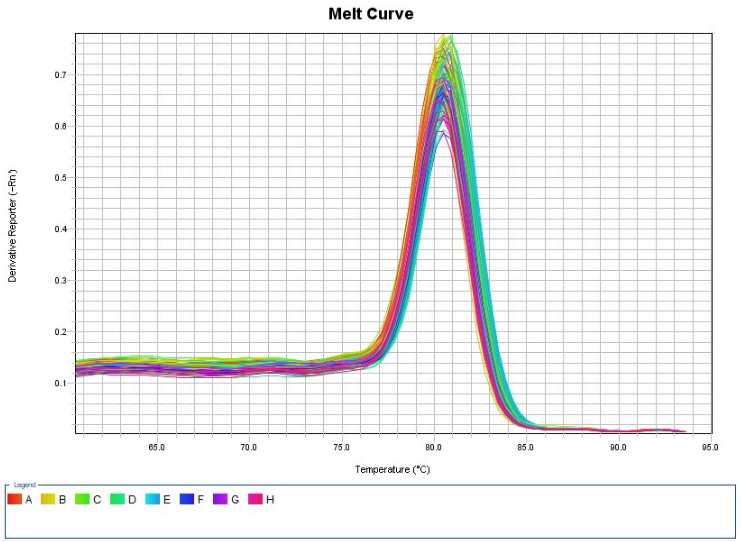

EcEF1-a

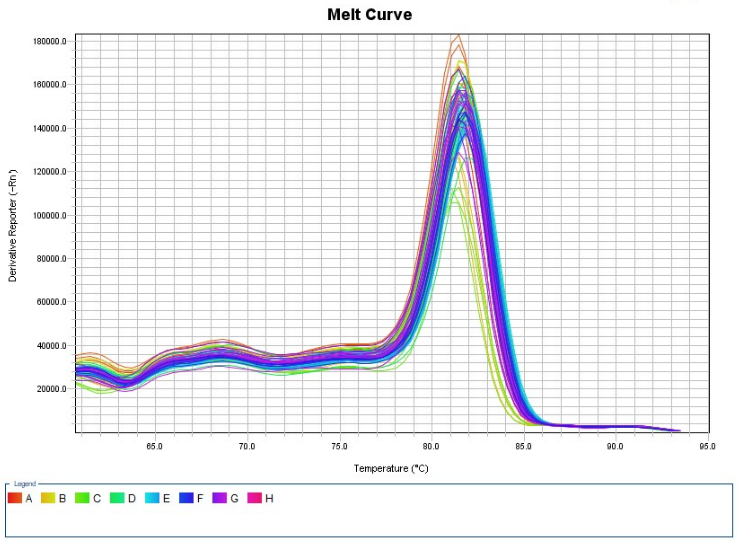

EcACT

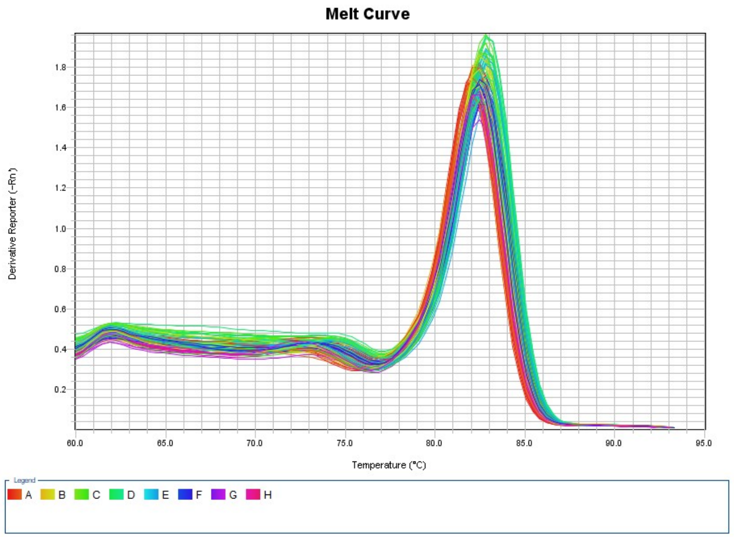

EcTUB

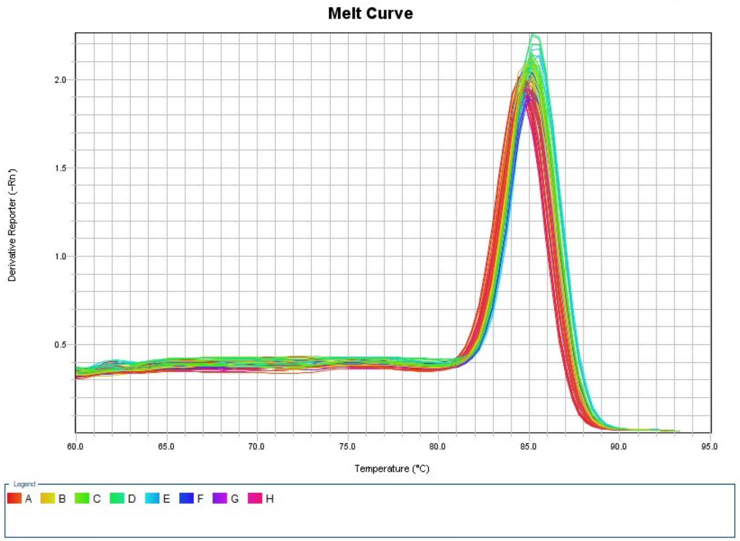

EcPTB

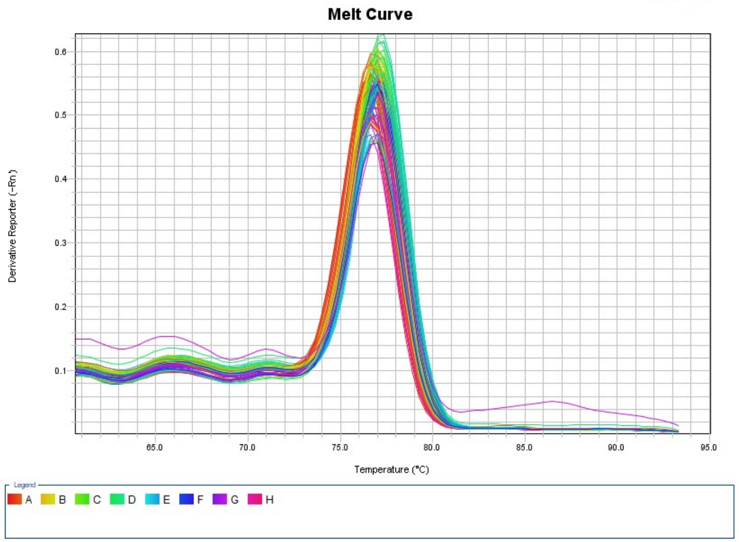

EcUBP6

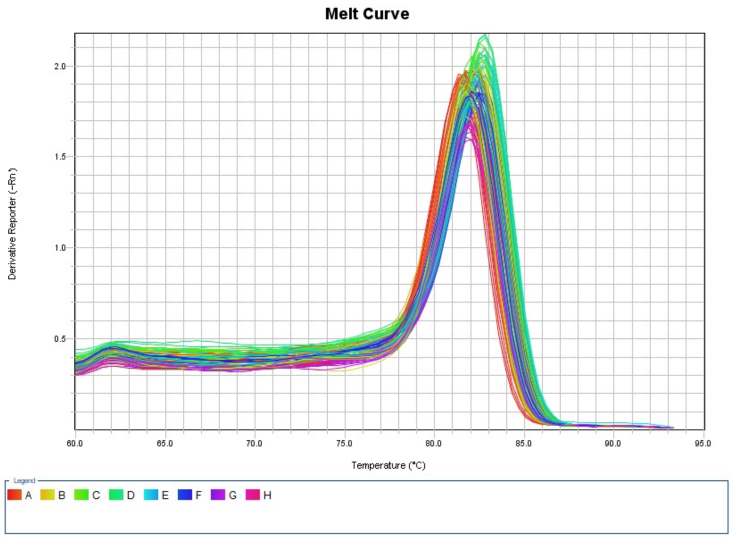

EcH2B

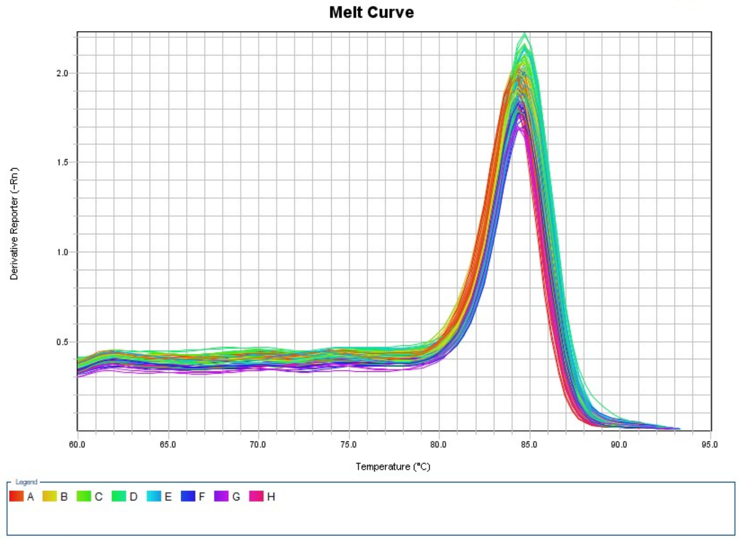

EcEUC12

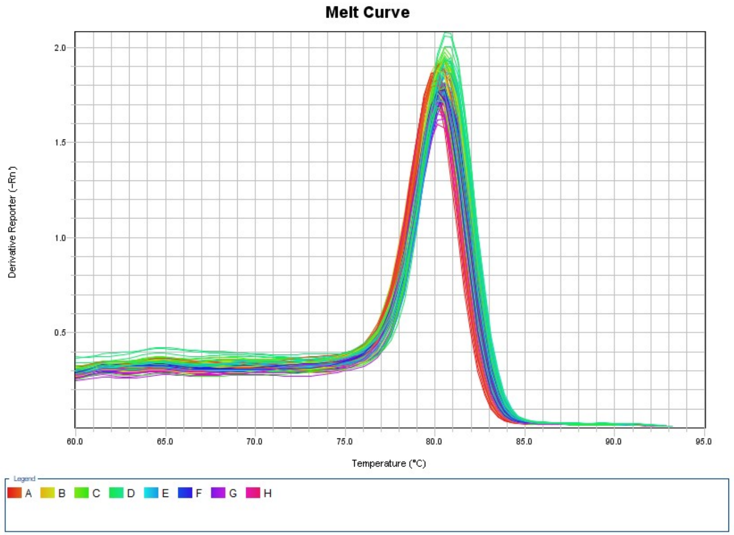

EcSAND

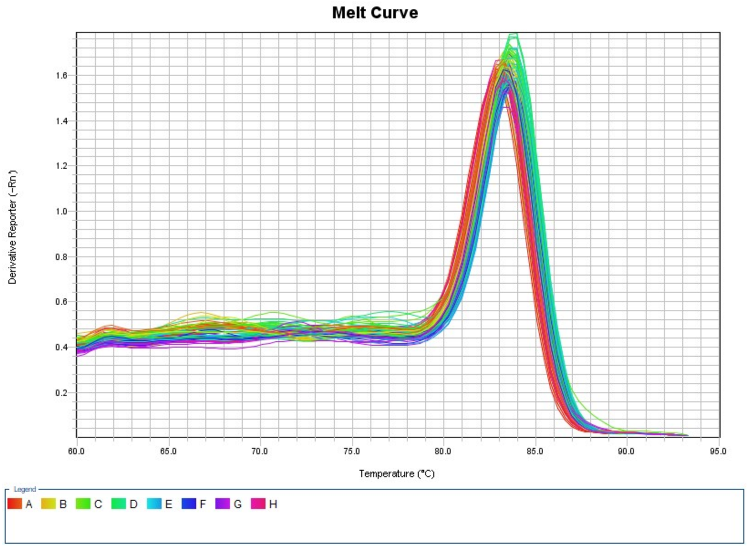

EcUPL7

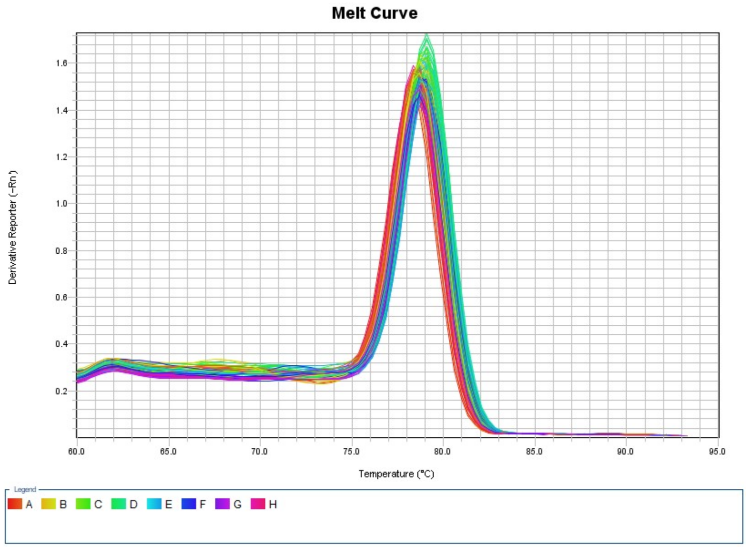

EcIDH

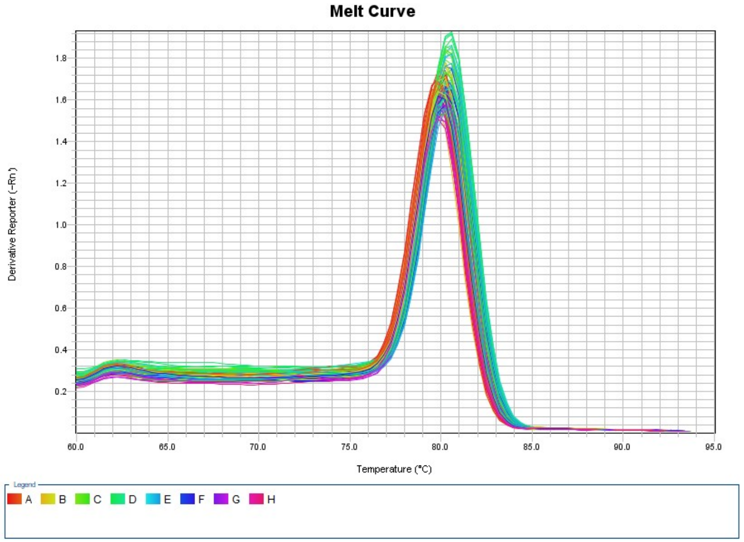

EcDREB2

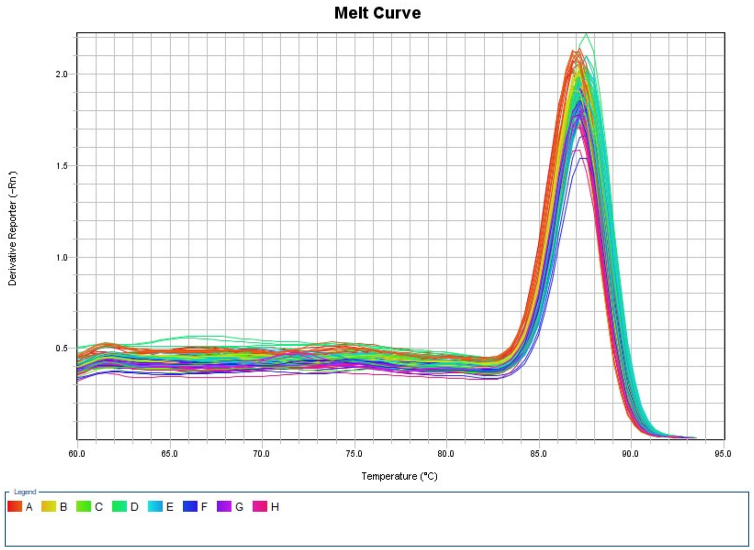

Supplement: Supplementary file 3 — Supplementary Information 3. [file 41598_2024_52948_MOESM3_ESM.pdf]
